# Calcium-Sensing Receptor Regulation of Gastrointestinal Hormone Secretion

**DOI:** 10.1210/endrev/bnaf040

**Published:** 2025-12-01

**Authors:** Javad Anjom-Shoae, Simon Veedfald, Arthur D Conigrave, Michael Horowitz, Christine Feinle-Bisset

**Affiliations:** Adelaide Medical School and Centre of Research Excellence in Translating Nutritional Science to Good Health, University of Adelaide, Adelaide, South Australia 5005, Australia; Department of Biomedical Sciences, University of Copenhagen, DK-2200 Copenhagen, Denmark; Charles Perkins Centre and School of Life and Environmental Sciences, University of Sydney, Sydney, New South Wales 2006, Australia; Adelaide Medical School and Centre of Research Excellence in Translating Nutritional Science to Good Health, University of Adelaide, Adelaide, South Australia 5005, Australia; Endocrine and Metabolic Unit, Royal Adelaide Hospital, Adelaide, South Australia 5000, Australia; Adelaide Medical School and Centre of Research Excellence in Translating Nutritional Science to Good Health, University of Adelaide, Adelaide, South Australia 5005, Australia

**Keywords:** CaSR, food intake, glycemia, gut hormones, L-phenylalanine, L-tryptophan

## Abstract

The interaction of dietary nutrients with chemoreceptors in the gastrointestinal tract after a meal stimulates the secretion of gut hormones, which trigger the key processes of digestion and absorption, and also regulate energy intake and postprandial glycemia. One of these receptors, first recognized for its capacity to gauge extracellular calcium (Ca^2+^), is the calcium-sensing receptor (CaSR). Subsequent to its cloning, the CaSR was found to sense not only Ca^2+^, but also L-amino acids (AAs) and, based on solved protein structures, distinct binding sites have been reported for Ca^2+^ ions and the aromatic AA, L-tryptophan (L-Trp). In the stomach and small intestine, the CaSR is expressed in enteroendocrine cells, and a substantial body of preclinical work has demonstrated that it mediates gut hormone secretion in response to L-Trp and another aromatic AA, L-phenylalanine (L-Phe), and that extracellular Ca^2+^ promotes these effects. In humans, intraluminal administration of L-Trp or L-Phe increases plasma levels of gut hormones, associated with reductions in both energy intake and the plasma glucose response to a subsequent meal. In addition, co-administration of Ca^2+^ enhances the effect of L-Trp to increase plasma levels of gut hormones (including cholecystokinin, glucagon-like peptide-1 and peptide YY) and reduce energy intake. These observations have implications for the development of novel nutrient-based management strategies for obesity and type 2 diabetes. This review considers preclinical and clinical evidence that CaSR activators, including extracellular Ca^2+^ as well as the aromatic AAs, L-Trp and L-Phe, stimulate gut hormones and lower both energy intake and postprandial glycemia.

## Essential Points

- The calcium-sensing receptor (CaSR), located on enteroendocrine cells, has the capacity to sense, and bind, both extracellular calcium (Ca^2+^) and aromatic L-amino acids (particularly L-tryptophan (L-Trp) and L-phenylalanine (L-Phe)), owing to its complex dimeric structure
- Preclinical studies have established that CaSR activation mediates the effects of Ca^2+^, L-Trp, and L-Phe on the secretion of gastrointestinal hormones, including gastrin, cholecystokinin (CCK), glucose-dependent insulinotropic polypeptide (GIP), glucagon-like peptide-1 (GLP-1), and peptide YY (PYY)
- L-Trp- or L-Phe-induced secretion of these hormones is augmented by increases in extracellular Ca^2+^ and is substantially reduced in the absence of extracellular Ca^2+^
- CaSR-mediated stimulation of GI hormones by L-Trp and L-Phe, or direct activation of the CaSR with CaSR agonists, has been shown to reduce food intake and lower postprandial plasma glucose in animals
- Recent clinical studies have demonstrated that Ca^2+^ also enhances the effects of L-Trp to stimulate CCK, GLP-1, and PYY secretion and suppress energy intake in humans
- Further research is needed to establish whether the CaSR mediates L-Trp-induced gut hormone release in humans, using CaSR antagonists, and to characterize the effects of selective agonists to evaluate their therapeutic potential

Dietary nutrients (eg, monosaccharides, L-amino acids (AAs), and fatty acids) activate specialized chemoreceptors that are expressed on gut hormone-producing (enteroendocrine) cells throughout the gastrointestinal (GI) mucosa, to stimulate the release of more than 20 hormones (1-3). While the precise sites of GI hormone release remain uncertain, specific regions of the GI tract are recognized as primary sites of their secretion. Accordingly, (i) gastrin is released primarily from the stomach, (ii) cholecystokinin (CCK) and glucose-dependent insulinotropic polypeptide (GIP) from the proximal small intestine, and (iii) glucagon-like peptide-1 (GLP-1) and peptide YY (PYY) from the distal small intestine (4), although there is now evidence for co-localization of multiple hormones within the same enteroendocrine cell type (5). The release of these hormones is central to the regulation of key gut functions, including gastric acid secretion, gastric emptying, intestinal digestion, and absorption of nutrients, as well as important systemic effects, including regulation of appetite, energy intake, and postprandial plasma glucose (6-8).

**Figure 1.**
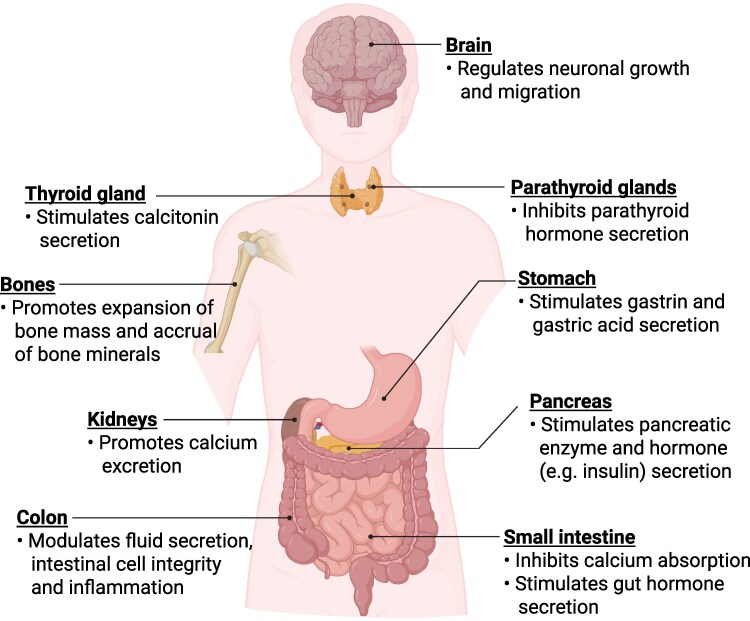
Physiological roles of the calcium-sensing receptor. Figure created with BioRender.com.

One key chemoreceptor that is expressed in the gut is the calcium-sensing receptor (CaSR). The CaSR was first cloned from a bovine parathyroid mRNA library in the early 1990s based on its capacity to sense extracellular divalent and trivalent cations (9), and it is now understood to be critical to Ca^2+^ homeostasis **(**Fig. 1**)** via (i) inhibitory control over parathyroid hormone (PTH) secretion from parathyroid chief cells and renal Ca^2+^ reabsorption; (ii) stimulatory control of calcitonin secretion from parafollicular C-cells of the thyroid glands; as well as (iii) a range of effects on bone cells (10). In the GI tract, the CaSR also exerts local inhibitory control on intestinal Ca^2+^ absorption (11). These discoveries led to the development of the currently used CaSR positive allosteric modulators, now widely used in the treatment of several hypercalcemic disorders, including various forms of primary and secondary hyperparathyroidism, typically caused by inactivating mutations and downregulation of the CaSR, respectively (12, 13). In addition, the therapeutic potential of CaSR negative allosteric modulators is being investigated for hypocalcemic disorders, such as autosomal dominant hypocalcemia (14, 15).

Shortly after the development of CaSR-expressing human embryonic kidney (HEK-293) cells as a convenient cell expression model for studies of the wild-type and mutant CaSRs (16), and in the presence of suprathreshold extracellular Ca^2+^ concentrations, the CaSR was unexpectedly found to act as a nutrient sensor for both aromatic and aliphatic AAs, of which L-tryptophan (L-Trp) and L-phenylalanine (L-Phe) were the most potent activators (17, 18). This work stimulated several predictions regarding the role of the CaSR as a multimodal sensor in the GI tract (19) and raised questions regarding the molecular basis by which 2 distinct classes of nutrients interact to promote receptor activation.

Subsequent preclinical studies, using CaSR inhibitors or genetically modified animal models (eg, CaSR knockout mice), provided direct evidence that L-Trp and L-Phe engage the CaSR to stimulate the secretion of several GI hormones, including gastrin (20, 21), CCK (22-26), GIP (25-27), GLP-1 (27-30), and PYY (27, 28, 30). Moreover, L-Trp- or L-Phe-induced secretion of these hormones was shown to be augmented by increases in extracellular Ca^2+^ (20-22, 24-27); in contrast, their stimulatory effects, particularly on CCK, GIP, and GLP-1, were compromised in vitro upon withdrawal of extracellular Ca^2+^ (22-24). CaSR-mediated stimulation of GI hormone release by these AAs, or in response to other activators, reduces food intake as well as the postprandial glucose response in several animal models (28, 30-33).

In humans, while the evidence for CaSR-mediated stimulation of Ca^2+^- or AA-induced GI hormone release is incomplete (although reported in human organoids (34, 35)), in part due to a paucity of studies using CaSR inhibitors, both Ca^2+^ and AAs have the capacity to stimulate the secretion of gastrin, GIP, GLP-1, and PYY, associated with reductions in energy intake and/or postprandial plasma glucose. For example, in 2 recent studies, intraluminal administration of Ca^2+^, in the form of a CaCl_2_ solution, increased plasma GLP-1 and PYY levels in both healthy individuals (36), and those with obesity (37). In some other studies, which have reported effects of Ca^2+^-containing meals on gut hormone secretion, however, the role of Ca^2+^ has been less clear, because it was primarily administered as a component of a test drink/meal (eg, milk or yogurt), in which other macronutrients were present (38-44), rather than as a defined supplement. There is also evidence that Ca^2+^ enhances the effect of aromatic AAs to increase plasma levels of gut hormones and to suppress energy intake. For example, in both healthy individuals and those with obesity, L-Trp-stimulated increases in CCK, GLP-1, and PYY secretion and reductions in energy intake were enhanced by the addition of Ca^2+^ (36, 37). Thus, in human studies, Ca^2+^ and AAs act together to stimulate GI hormone release, consistent with the 2-site engagement of the CaSR described in preclinical studies. They also negatively modulate energy intake and lower postprandial plasma glucose.

This review provides an overview of the outcomes of preclinical and clinical studies regarding the role of the CaSR in mediating the release of gut hormones in response to Ca^2+^ in the absence, or presence, of aromatic AAs. Although other AAs (eg, L-tyrosine, L-histidine, L-alanine, L-threonine or analogues of L-arginine (17, 45, 46)) also engage the CaSR, we focus herein on the effects of 2 aromatic AAs, L-Trp and L-Phe, which are the most potent AA activators in HEK-293 cells (17) and human parathyroid cells (47). Some studies were designed to test whether the CaSR is the molecular target of Ca^2+^ and AAs, either alone or in combination, with respect to GI hormone release (eg, with the use of selective CaSR inhibitors). Other studies have solely focused on whether Ca^2+^ or AAs, or a combination of Ca^2+^ and AAs, were effective at elevating plasma GI hormone levels, as would be predicted for cell types that express the CaSR protein. This review considers both types of studies and also the impacts of the CaSR activators, L-Trp and L-Phe, in the absence and presence of Ca^2+^, on GI hormone levels in humans (48-63), and whether the 2 distinct nutrient classes of CaSR ligands interact positively to reduce energy intake and lower postprandial plasma glucose profiles.

In considering the effects of aromatic AAs, it is important to note that a distinct class A G protein–coupled receptor, known as GPR142, has been de-orphanized and found to be expressed in some, but not all, enteroendocrine cells (34). While this receptor responds to L-Trp, L-Phe, and L-tyrosine, it does not appear to respond more broadly to AA mixtures that arise from protein digestion (46, 64), or in a Ca^2+^-dependent manner, as observed for the CaSR. While this review focuses on the CaSR for the reasons stated above, we also draw attention to settings in which GPR142 may contribute to GI hormone release, particularly in the case of GIP.

## The Calcium-Sensing Receptor

The CaSR was first cloned from a bovine parathyroid mRNA library (9) and, subsequently, from rat kidney (65) and human adenomatous parathyroid (66) mRNA in the early-mid 1990s. The mRNA encodes a class C G protein–coupled receptor (GPRC2A), which forms homodimers when expressed in cell membranes (67-69). In humans, each subunit is composed of 1078 AA residues, taking the form of an N-terminal extracellular region (formed from a venus fly trap [VFT] domain, a cysteine-rich domain [CRD], and a C-terminal linker), a heptahelical bundle (ie, a 7-transmembrane domain unit), and an intracellular C-terminal domain (67-69). The intracellular loops (iCLs), particularly iCL2 and iCL3, in collaboration with the intracellular C-terminus, couple intracellularly to several G proteins, including G_q/11_, G_i_, G_0_, and, in some cases, Gs, and thereby mediate cellular signal transduction pathways (67-69). This structure provides the CaSR with diverse binding sites to respond to a surprisingly extensive list of activators which, in addition to its principal agonist, Ca^2+^, include (i) divalent cations, such as Mg^2+^, and trivalent cations, such as Gd^3+^ (70); (ii) natural positive modulators that bind in the VFT domain (eg, AAs (17, 71), γ-glutamyl peptides (72, 73), and spermine (74)); and (iii) high-potency synthetic positive allosteric modulators (eg, cinacalcet and NPS-R568) and negative allosteric modulators (eg, Calhex231 and NPS-2143) (75). In addition to these pharmacological activators and inhibitors, receptor activity is also promoted by low ionic strength and high pH (76, 77). It is now known that the receptor undergoes repetitive transitions between 2 main states, an inactive and an active state (67-69). These 2 states impact the dimer interface at the level of the VFT domains, proximal CRDs, and heptahelical bundle and act to either facilitate or obstruct the creation of a single G protein binding site per dimer formed by the intracellular loops, transmembrane domain-3, and proximal C-terminus. Agonists and positive modulators promote the active state, while antagonists and negative modulators promote the inactive state.

### Structural Basis of Ligand Binding and Activation of the CaSR

Recent studies have provided critical insights into the nature of the CaSR's distinct binding sites for Ca^2+^ and AA, and the mechanism(s) by which they interact. Thus, several solved (ie, high-resolution) structures of CaSR homodimers have been reported in both inactive and active states, in the presence of low and high Ca^2+^, and in the absence and presence of L-Trp (67-69). These studies have led to the identification of a single AA binding site per subunit of the CaSR, which is located in the bilobed cleft of the receptor's N-terminal VFT domain. This corresponds to the canonical class C agonist binding site for the AA, glutamate, in metabotropic glutamate receptors and the AA analogue, γ-aminobutyrate, in GABA_B_ receptors (78). The structure studies have also led to the identification of 4 Ca^2+^ binding sites per subunit. Of these sites, “Site-4,” which is located at the extreme C-terminus of the VFT and at the N-terminus of the CRD, which follows it in tandem, stabilizes the active form of the receptor by electrostatic interactions across the dimer interface. As the receptor's dimer interface extends from VFT Lobe-1 to include VFT Lobe-2 upon activation, the extreme C-termini of the dimeric CRDs are drawn toward each other. Because the CRDs are rigid, caliper-like structures, this has the effect within the membrane of apposing the pair of 7-transmembrane domain units and inducing mutual rotations to reposition TM helices 6 and 7 at the dimer interface, leading to the formation of a single G protein binding site per receptor dimer. Thus, L-Trp, and presumably other AA molecules, promote closure of the bilobed cleft of the receptor's VFT and induce extended apposition of the dimeric subunits involving VFT Lobe-2 and the CRD. In addition, Ca^2+^ interacts positively with the AA-bound (VFT closed) form of the receptor by stabilizing the extended dimer interface.

### Physiological Roles of the CaSR

The CaSR plays a role in Ca^2+^ homeostasis, in which it acts to lower the circulating Ca^2+^ concentration (10), as well as in energy metabolism, where it promotes digestion and absorption of nutrients, and, via downstream effects, modulates plasma nutrient concentrations and food intake (17-19). With respect to its critical role in Ca^2+^ homeostasis, the CaSR is highly expressed in chief cells of the parathyroid glands, where it mediates extracellular Ca^2+^-induced feedback inhibition of PTH secretion (79), and in cortical thick ascending limb cells of the renal tubules, where it mediates extracellular Ca^2+^-dependent inhibition of Ca^2+^ reabsorption, to promote urinary Ca^2+^ excretion (80). These 2 key effects to lower circulating Ca^2+^ are supported by the expression of the CaSR in thyroid parafollicular C-cells, which mediates extracellular Ca^2+^-stimulated calcitonin release to inhibit bone resorption and promote bone formation (81). In addition, the CaSR is expressed in cells that regulate skeletal turnover, including bone-resorbing osteoclasts and bone-forming osteoblasts, and thereby favors an expansion of bone mass and accrual of bone mineral (82).

Although expressed at low levels in intestinal epithelial cells, when compared with its levels in the parathyroid, the renal thick ascending limb, and indeed enteroendocrine cells, the CaSR mediates local feedback control of Ca^2+^ absorption from luminal contents, whereby elevations in circulating Ca^2+^ on the contraluminal (ie, blood-facing) surface of intestinal epithelial cells inhibit intestinal Ca^2+^ absorption (11).

The CaSR is also expressed in organs and tissues that are not associated with Ca^2+^ metabolism, including the brain, liver, pancreas, and enteroendocrine cells, indicative of potential roles in other physiological processes (83). Beyond its physiological role, CaSR expression can also be altered in various benign and malignant tumors, including parathyroid, breast, prostate, and colon cancers, where it may contribute to tumorigenesis (84). However, discussion of CaSR overexpression, and/or its oncogenic or tumor-suppressive roles, is beyond the scope of the present review. In the GI tract, as we will discuss in detail, the CaSR acts as a nutrient sensor, particularly for AAs and Ca^2+^ (85). Its expression in enteroendocrine cells (86) enables its involvement in the release of gut hormones that impact energy metabolism in several phases. In the initial phase of the gut hormone response, the release of digestive enzymes is stimulated, leading to the breakdown of macronutrients and, subsequently, the absorption of their building blocks, ie, AAs, monosaccharides, and fatty acids. In the subsequent phases of the response, impacts are exerted on plasma nutrient profiles (eg, postprandial glucose levels) and regulation of appetite. Thus, due to its expression across gastric parietal cells (20, 87), pancreatic acinar (88), beta (33) and alpha (89) cells, and hepatocytes (90), the CaSR directly couples nutrient signals to gastric acid secretion, pancreatic enzyme, hormone (eg, insulin and glucagon), and bile secretion. In the case of pancreatic islet beta and alpha cells (89, 91), the CaSR operates alongside the second aromatic AA sensing receptor, GPR142, which is expressed at high levels in these cell types (92). The CaSR has also been reported to modulate the integrity of the intestinal epithelium (93), fluid secretion (94), and local inflammatory responses (95).

### Role of the CaSR in Mediating Nutrient-Dependent GI Hormone Secretion

**Figure 2.**
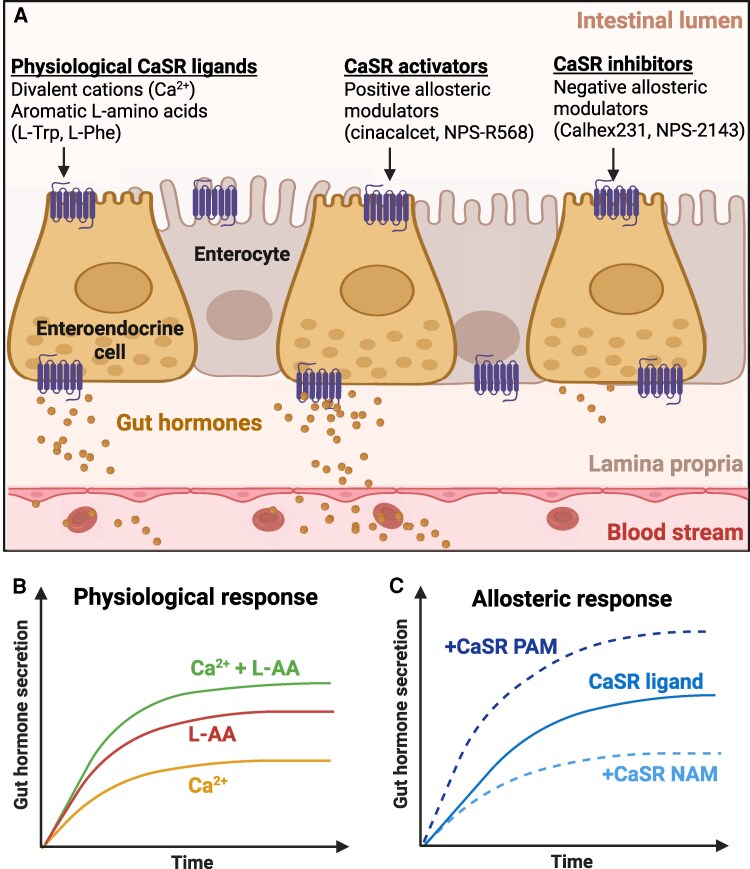
(A) In the gastrointestinal (GI) tract, the calcium-sensing receptor (CaSR) is expressed on both the apical and basolateral membranes of enteroendocrine cells to mediate the effects of physiological CaSR ligands, including extracellular Ca^2+^ and the aromatic L-amino acids (AAs), L-tryptophan (L-Trp), and L-phenylalanine (L-Phe), on the stimulation of GI hormones. The CaSR also responds to synthetic positive allosteric modulators (PAMs; eg, cinacalcet and NPS-R568) and negative allosteric modulators (NAMs; eg, Calhex231 and NPS-2143), which stimulate and inhibit GI hormone secretion, respectively. (B) The physiological response of the CaSR to either Ca^2+^ or AAs to stimulate GI hormones has been shown to be enhanced when the 2 ligands are administered in combination. (C) In addition, the stimulation of GI hormones by physiological CaSR ligands is allosterically augmented by CaSR PAMs and diminished by CaSR NAMs. Figure created with BioRender.com.

Preclinical findings, based on cell culture and animal models, have established a role for the CaSR in the nutrient-dependent stimulation of GI hormone release (20-30) (Table 1). Initial studies demonstrated that the CaSR mediates extracellular Ca^2+^-stimulated gastrin secretion (87), and there was also evidence for CaSR protein expression in acid-secreting parietal cells and pepsinogen-secreting mucous cells of gastric glands (96). Following the recognition that the CaSR also senses AAs, particularly of the aromatic subgroup (17, 18), roles for the CaSR in mediating AA-induced secretion of other GI hormones, including CCK, GIP, GLP-1, and PYY, have been reported (17-27). The finding that the CaSR is expressed on both the apical and basolateral membranes of enteroendocrine cells suggests that it responds to changes in the composition of both the luminal and contraluminal fluids to stimulate GI hormone release (19). It was also demonstrated that the addition of extracellular Ca^2+^ enhanced the stimulation of GI hormones by aromatic AAs (20-22, 24-27) (Fig. 2). While the role of the CaSR in mediating Ca^2+^- or AA-induced stimulation of GI hormones has not been investigated in humans, several studies have reported that administration of CaSR agonists (97, 98), oral Ca^2+^ supplements (99), or intravenous (IV) infusions of Ca^2+^, sufficient to raise plasma total Ca^2+^ concentration from approximately 2.2-2.4 mmol/L to 2.8-3.0 mmol/L (100), acutely increase plasma gastrin levels. In healthy individuals, in the case of IV infusion of Ca^2+^, plasma gastrin levels rose by 20% and gastric acid secretion by 40% (100). However, these effects in healthy individuals were not observed in patients with primary hyperparathyroidism, who characteristically have elevated plasma levels of gastrin as well as Ca^2+^ (99, 101). Interestingly, the CaSR positive modulator cinacalcet was reported to have no effect on plasma gastrin or CCK levels in patients with hyperparathyroidism (102), possibly because (i) the gastrin and CCK levels were already raised in response to the high prevailing plasma Ca^2+^ concentration; (ii) desensitization of the CaSR in enteroendocrine cells upon chronic exposure to high Ca^2+^; or (iii) the 2-hourly blood sampling protocol used in the study was most likely insufficient to detect short-term changes in plasma CCK, which usually occur during the first 60 minutes.

**Table 1.** Preclinical studies on the role of the calcium-sensing receptor in the effects of aromatic L-amino acids on gut hormone secretion

Experimental model	AA type	Gut hormones	Treatment				Ref
			AA alone	AA + CaSR activator	AA + CaSR inhibitor	AA + extracellular Ca^2+^	
STC-1 cells	L-Phe	CCK	↑	NR	↓	↑↑	(22)
CaSR-null mice (in vivo)	L-Phe	Gastrin	↑	↑↑	↓	↑↑	(20)
Duodenal tissue (mice)	L-Trp	CCK	↑	NR	↓	NR	(23)
	L-Phe	CCK	↑		↓		
Duodenal tissue (mice)	L-Phe	CCK	↑	NR	NR	↑↑	(24)
Intestinal tissue (rats)	L-Trp	GIP	↑	↑↑	↓	↑↑	(27)
		GLP-1	↑	↑↑	↓	↑↑	
		PYY	↑	↑↑	↓	↑↑	
	L-Phe	GIP	↑	↑↑	↓	↑↑	
		GLP-1	↑	↑↑	↓	↑↑	
		PYY	↑	↑↑	↓	↑↑	
Oral administration (rats, mice) (in vivo)	L-Phe	GLP-1	↑	NR	↓	NR	(28)
		PYY	↑		↓		
Intestinal tissue (pig)	L-Trp	Gastrin	↑	↑↑	↓	↑↑	(21)
	L-Phe	Gastrin	↑	↑↑	↓	↑↑	
Intestinal tissue (pig)	L-Trp	CCK	↑	NR	↓	↑↑	(25)
		GIP	↑		↓	↑↑	
STC-1 cells	L-Phe	GLP-1	↑	NR	↓	NR	(29)
Intestinal tissue (pig)	L-Phe	CCK	↑	NR	↓	↑↑	(26)
		GIP	↑		↓	↑↑	
Intraduodenal infusion (rats) (in vivo)	L-Trp	GLP-1	↑	NR	↓	NR	(30)
		PYY	↔		↔		

Abbreviations: AA, amino acid; Ca^2+^, calcium; CaSR, calcium-sensing receptor; CCK, cholecystokinin; GIP, glucose-dependent insulinotropic polypeptide; GLP-1, glucagon-like peptide-1; NR, not reported; PYY, peptide YY.

### Cellular Mechanisms Linking CaSR Activation by AAs to Secretion of Hormones From Enteroendocrine Cells

In the proximal small intestine, luminal glucose triggers an early phase of GLP-1 and GIP release, and in turn, insulin secretion, dependent upon luminal membrane expression of sodium-glucose cotransporter 1 (SGLT1) in enteroendocrine L and K cells, respectively. The key events appear to be (i) glucose-dependent Na^+^ influx; (ii) depolarization and initiation of action potentials; and (iii) Ca^2+^ influx via voltage-gated Ca^2+^ channels (103). Exocytosis of hormone-containing large, dense-core, secretory vesicles follows via SNARE-mediated fusion with the plasma membrane (3). The identities of the Ca^2+^-binding proteins that promote fusion and exocytosis are not yet clear, but may include synaptotagmin-7, which is (i) expressed in enteroendocrine cells; (ii) regulated via high-affinity Ca^2+^-binding sites, like other canonical synaptotagmins; and (iii) required for glucose-dependent GLP-1 secretion (104).

The CaSR, on the other hand, is expressed primarily on the contraluminal (blood-facing) membranes of enteroendocrine cells, including gastrin-secreting G cells (87) and CCK-secreting I cells (23). Thus, the CaSR, like other nutrient-sensing receptors, is positioned to respond to protein-derived oligopeptides and AAs following intestinal absorption. The mechanisms by which dietary protein induces the secretion of GLP-1 and GIP are different to those described above for carbohydrates. In particular, the most powerful mechanisms for protein-induced GLP-1 and GIP secretion depend on the uptake of oligopeptides and free AAs across the luminal membranes of intestinal epithelial cells, followed by intracellular catabolism of oligopeptides to free AAs and the release of AAs across the basolateral membranes onto the interstitial surfaces of enteroendocrine cells. Thus, recent analyses using a vascularly-perfused rat proximal intestine model concluded that secretion of GLP-1 in response to either ingested peptone (a meat-derived extract rich in oligopeptides), or to mixtures of AAs, is dependent primarily upon absorption by intestinal epithelial cells, followed by CaSR-mediated detection on the blood-facing membranes of enteroendocrine cells (46, 64).

**Figure 3.**
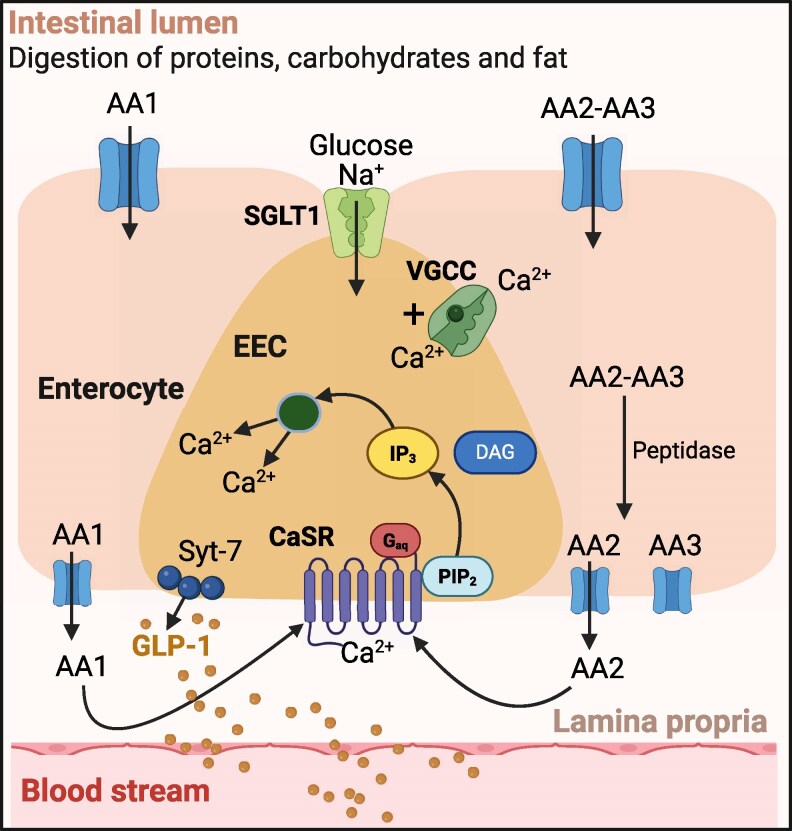
Intracellular signaling cascade that evokes GLP-1 secretion in response to L-amino acid activation of the CaSR. Ingested protein is digested in the small intestinal lumen. Resulting free amino acids (AA1) and oligopeptides (eg, the dipeptide AA2-AA3) are taken up by specific transporters on the apical (luminal) membrane of intestinal epithelial cells. In the case of oligopeptides, cytoplasmic peptidases release free AAs, which are delivered into the interstitial space. There, AAs bind and activate CaSRs located on the basolateral membrane of enteroendocrine cells (EECs). The AA-bound CaSR elevates cytoplasmic free Ca^2+^ concentration via release from intracellular stores. The intracellular Ca^2+^ signal triggers exocytosis of GLP-1 (as well as other gut hormones)-containing secretory vesicles. Also shown is glucose-dependent Na^+^ uptake across the luminal membrane of EECs, which induces depolarization, the transmission of action potentials and Ca^2+^ influx via voltage-gated Ca^2+^ channels (VGCCs). Free fatty acids are also transported across intestinal epithelial cells to act on basolateral FFA receptors, eg, FFAR1 (not shown). Figure created with BioRender.com.

After delivery of AAs from the GI lumen to the intestinal interstitium, a subset of available AAs bind to the CaSR on the basolateral membranes of enteroendocrine cells **(**Fig. 3**)**. As described above, aromatic AAs, including L-Trp and L-Phe, stabilize the active form of the CaSR's dimeric VFT, provided the extracellular Ca^2+^ concentration is sufficient (≥0.75 mM) to stabilize the dimer interface at the N-terminus of the receptor's Cys-rich domains. At higher interstitial Ca^2+^ concentrations (eg, from 1.2-1.5 mM), additional AAs are recruited to stabilize the active forms of more receptor molecules. After activation by AAs in the presence of extracellular Ca^2+^, the CaSR recruits diverse G proteins, including G_q_ and G_i_. With respect to the secretion of gut hormones, the key step appears to be G_αq_-dependent activation of PI-PLC with attendant generation of inositol 1,4,5-trisphosphate (IP_3_) and, in turn, Ca^2+^ release from ER membrane-bound intracellular stores to raise the intracellular Ca^2+^ concentration. Consistent with this, AAs induced elevations in intracellular Ca^2+^ (23, 35) and AA-dependent activation of GLP-1 release from the perfused rat intestine was markedly suppressed not only by vascular delivery of the CaSR inhibitor NPS-2143, but also by the PI-PLC inhibitor, U73122 (64). Whether the CaSR might also stimulate the secretion of gut hormones by raising the cytoplasmic cAMP concentration (103) either by coupling to G_s_ (105) or via G_βγ_-dependent activation of the adenylyl cyclase isoform, ADCY2, as described for the free FA receptor FFAR1 (106), is unknown.

## Effects of Ca^2+^ on GI Hormone Secretion, Energy Intake, and Blood Glucose

Ca^2+^-dependent stimulation of gastrin and gastric acid secretion has been recognized since the first half of the twentieth century (107). In contrast, the ability of Ca^2+^ to stimulate the secretion of other GI hormones, particularly those involved in the regulation of energy intake and blood glucose, including CCK, GIP, GLP-1, and PYY, has only received attention more recently. The following sections review the current understanding of the effects of Ca^2+^ on GI hormone secretion, energy intake, and blood glucose, from both preclinical and clinical studies.

### Preclinical Outcomes

The recognition that intracellular Ca^2+^ serves as an essential messenger to mediate secretion of GI hormones has led to a particular interest in understanding the role of extracellular Ca^2+^ in this process (108-110). It is now well established, in both in vitro and in vivo studies, that extracellular Ca^2+^ plays a key role in the stimulation of gastrin and gastric acid secretion (111-118). For example, the removal of extracellular Ca^2+^ by different Ca^2+^-chelating agents (eg, EDTA or EGTA) diminished the stimulation of gastrin and gastric acid secretion (111, 114), while IV infusion of Ca^2+^ increased serum gastrin levels and gastric acid secretion (115). Subsequent studies provided evidence that extracellular Ca^2+^ might be critical in the secretion of other GI hormones (119-127). For example, stimulation of CCK secretion by different stimuli, including bombesin, an agent that increases intracellular Ca^2+^ levels (119, 122), monitor peptide (121), peptones (124), fatty acids (123, 127), aldehydes (125) or endogenous CCK-releasing factor (126), was reduced, if not totally inhibited, in the absence of extracellular Ca^2+^, achieved by using either EDTA or EGTA and/or voltage-gated Ca^2+^ channel blockers. In addition, both basal and/or glucose-induced secretion of GLP-1 from cell lines (128, 129) or a perfused rat intestine model (130) was inhibited by Ca^2+^ channel blockers. While these findings indicate the importance of extracellular Ca^2+^ in the stimulation of GI hormones, the precise underlying cellular mechanisms, particularly whether this effect involves the CaSR, or is achieved through other pathways, such as voltage-gated Ca^2+^ channels, which are highly expressed on enteroendocrine cells and underlie Ca^2+^ influx into the cells, remain to be fully understood (103).

A role for the CaSR in the stimulation of GI hormone secretion was first demonstrated in the late 1990s in in vitro studies, in which an elevation of the extracellular Ca^2+^ concentration or exposure to the CaSR activator spermine stimulated gastrin secretion from human antral G cells (87, 131, 132). In a subsequent in vivo study in transgenic mice, oral administration of Ca^2+^ or the CaSR agonist cinacalcet stimulated gastrin and gastric acid secretion in wild-type, but not CaSR-null, mice (20). In rats, oral Ca^2+^-induced stimulation of PYY secretion was reduced by pretreatment with the CaSR inhibitor, NPS-2143, by about 44% (31).

There is also preclinical evidence indicating that activation of the CaSR reduces food intake and/or blood glucose levels (31, 33). For example, in rats, oral administration of cinacalcet reduced food intake (31). Furthermore, IV administration of the CaSR agonist NPS-R568 increased plasma insulin and lowered postprandial blood glucose levels, whereas the CaSR inhibitor NPS-2143 blocked these effects (33). There have been no studies investigating the involvement of the CaSR in the effects of Ca^2+^ on food intake or blood glucose, but a number of animal studies indicate a link between dietary Ca^2+^ intake and energy metabolism, with high-Ca^2+^ diets potentially attenuating weight gain, at least in part, by simultaneously stimulating lipolysis and inhibiting lipogenesis during caloric restriction (133-135). Thus, further studies are required to clarify the role of the CaSR in this context.

### Clinical Outcomes

As alluded to earlier, in humans, Ca^2+^ has long been known to increase plasma gastrin levels and gastric acid secretion (99, 101). However, its effect on other GI hormones is less clear. The majority of the available studies that investigated the acute or longer-term effects of Ca^2+^ have done so by incorporating Ca^2+^ in a meal or drink, that is, along with other nutrients, such as protein (Table 2) (38-44). For example, several studies used dairy products, which are also rich in whey and casein, known to stimulate GI hormone secretion (7). In contrast, few studies have evaluated the effects of Ca^2+^, in an isolated form, on gut hormone secretion (36, 37). Moreover, the duration of the Ca^2+^ intervention and the characteristics of the study population (eg, healthy lean, overweight, or with obesity), are also likely to be relevant. Outcomes of the different approaches are reviewed in the following sections.

**Table 2.** Effects of calcium on plasma concentrations of gut hormones, energy intake, and plasma glucose in humans

Study population	*N*	Age (y)	BMI (kg/m^2^)	Dose of calcium (mg)	Type of intervention	Duration	Outcomes			Ref
							Gut hormones	Energy intake (kcal)	Plasma glucose (mmol/L)	
Healthy overweight	18 (M)	25 ± 1	27 ± 1	850	Oral mixed-nutrient meal	7 h	↔ CCK	↔	↔	(38)
							↑GLP-1			
							↔ PYY			
Metabolic syndrome	38 (M/F)	52 ± 2	32 ± 1	1400/day	Oral mixed-nutrient meal	12 w	↔ GIP	↔	↔	(41)
							↑GLP-1			
							↑PYY			
Healthy lean	10 (M)	25 ± 3	26 ± 2	1200	Oral mixed-nutrient meal	4 h	↑GIP	NR	↔	(39)
							↑GLP-1			
Healthy lean	13 (M)	26 ± 4	24 ± 1	1000/day	Oral supplement	2 w	↔GIP	NR	NR	(43)
							↔GLP-1			
Healthy lean	9 (M)	27 ± 4	23 ± 2	1100/day	Oral mixed-nutrient meal	3 w	↔GIP	NR	↔	(42)
							↑GLP-1			
Healthy lean	20 (M/F)	22 ± 1	22 ± 1	1170	Oral mixed-nutrient meal	1 h	↔GIP	↔	NR	(44)
							↔GLP-1			
Healthy lean	20 (M/F)	25 ± 4	23 ± 3	1000	Oral mixed-nutrient meal	2 h	↑GIP	NR	↔	(40)
							↑GLP-1			
							↑PYY			

Abbreviations: BMI, body mass index; CCK, cholecystokinin; F, female; GIP, glucose-dependent insulinotropic polypeptide; GLP-1, glucagon-like peptide-1; M, male; NR, not reported; PYY, peptide YY; y, years.

#### Effects of acute (single-dose) dietary Ca^2+^ on GI hormones, energy intake, and blood glucose

Several studies have investigated the effects of dietary Ca^2+^ on the plasma levels of several GI hormones in healthy individuals. In one such study, consumption of a breakfast consisting of a porridge prepared with milk and supplemented with milk-extracted Ca^2+^ powder (Ca^2+^ dose: 1200 mg) resulted in higher postprandial levels of plasma GIP, GLP-1, and insulin, when compared with a porridge prepared with water (Ca^2+^ dose: 248 mg) (39). A second study compared the effects of a porridge prepared with water and supplemented with either 1170 mg milk-extracted Ca^2+^ or 25 g protein, or both, with the effects of a porridge that contained much lower amounts of Ca^2+^ (104 mg) and protein (4 g) (44). While the porridge, when supplemented with Ca^2+^ plus protein or with protein alone, increased plasma GLP-1 levels, the porridge that was enriched with Ca^2+^ alone had no effect (44), and none of the meals affected plasma GIP levels. A third study compared the effects of aqueous drinks, containing either 1000 mg of added Ca^2+^ in the form of calcium citrate or milk-extracted Ca^2+^ powder, or 50 g whey protein, or both Ca^2+^ and protein, on the stimulation of plasma GIP, GLP-1, and PYY (40). Interestingly, the drink that contained a combination of milk-extracted Ca^2+^ as well as protein resulted in significantly greater stimulations of GIP, GLP-1, and PYY, when compared with the effects of calcium citrate alone, or milk-extracted Ca^2+^ alone, protein alone, or water alone (40). When compared with water, ingestion of milk-extracted Ca^2+^ alone modestly increased plasma GLP-1 (40), while no effects were observed on plasma GIP or PYY levels.

A few studies have also investigated whether Ca^2+^-enriched foods acutely modulate energy intake (38, 44, 136, 137) and/or plasma glucose levels (38-40). For example, in one study, consumption of 600 mL skim milk, containing 1000 mg Ca^2+^, compared with 600 mL fruit juice, as part of a fixed-energy breakfast, induced greater postprandial fullness and was associated with a mean 8.5% reduction in energy intake at a subsequent ad-libitum lunch (136). In another study, in which healthy lean individuals consumed a porridge supplemented with either 1170 mg milk-extracted Ca^2+^ or 25 g protein, or both, only the porridge supplemented with both Ca^2+^ and protein significantly reduced ad-libitum energy intake by 169 kcal (17%), while smaller reductions in energy intake in response to Ca^2+^ alone or protein alone were not statistically significant (44).

The glucose-lowering effect of increased Ca^2+^ intake, if any, appears modest, and may be due to the effects of co-ingested nutrients. For example, in healthy individuals with overweight, isocaloric test meals, containing either 68, 350, or 793 mg of Ca^2+^ from dairy products, or 850 mg of Ca^2+^ in the form of a CaCO₃ supplement, had no effect on postprandial plasma glucose (38). Furthermore, no differences were evident in the plasma glucose response to a porridge containing either 1200 mg or 248 mg Ca^2+^ (39). In contrast, consumption of a drink, containing 1000 mg milk-extracted Ca^2+^ and 50 g whey protein, reduced postprandial plasma glucose (ie, primarily 60 minutes post-meal) more than drinks containing 1000 mg of milk-extracted Ca^2+^ or calcium citrate alone, although the absolute difference in plasma glucose was only 0.3 to 0.4 mmol/L (40).

#### Effects of longer-term Ca^2+^ supplementation on GI hormones, energy intake, and blood glucose

Several studies have investigated the longer-term effects of Ca^2+^ to evaluate whether the effects on GI hormones and/or energy intake, or plasma glucose, are sustained (41-43). For example, in healthy lean participants, consumption of bread enriched with 1100 mg Ca^2+^ daily for 3 weeks, as part of a controlled diet, significantly increased postprandial plasma levels of GLP-1, 30 minutes after the meal, more than non-enriched bread (42), although there were no significant effects on GIP, insulin, or postprandial glucose. Fasting GLP-1 concentrations were not affected by Ca^2+^ (42), suggesting that its primary effect is on meal-related GI hormone secretion. In another study in individuals with metabolic syndrome, supplementation of an energy-restricted diet with 1400 mg/day of Ca^2+^ from dairy products for 12 weeks had a greater stimulatory effect on postprandial plasma levels of GLP-1 and PYY, but not GIP, than 700 mg/day of Ca^2+^ (41), although no effects on energy intake or postprandial plasma glucose levels were evident. Consistent with these observations, the CaSR has been reported to be involved in the secretion of GLP-1 and PYY (28, 34), while secretion of GIP appears to involve GPR142 as well as the CaSR (35).

A few studies have reported the effect of Ca^2+^ supplementation on appetite perceptions, although not consistently and mostly under conditions of energy restriction, and findings have been inconclusive (137-140). For example, in a 6-month energy-restricted weight loss program in overweight women, Ca^2+^ supplementation in a dose of 1000 mg/d in the form of milk, reduced fasting levels of desire to eat and hunger, compared with a placebo supplement (140). A number of variables, including particularly the different nutritional composition of these foods, which may affect both the bioavailability and palatability of Ca^2+^ and, accordingly, its impact on GI hormone release, limit the ability to interpret the findings.

#### Effects of isolated Ca^2+^ on GI hormone secretion, energy intake, and blood glucose

**Figure 4.**
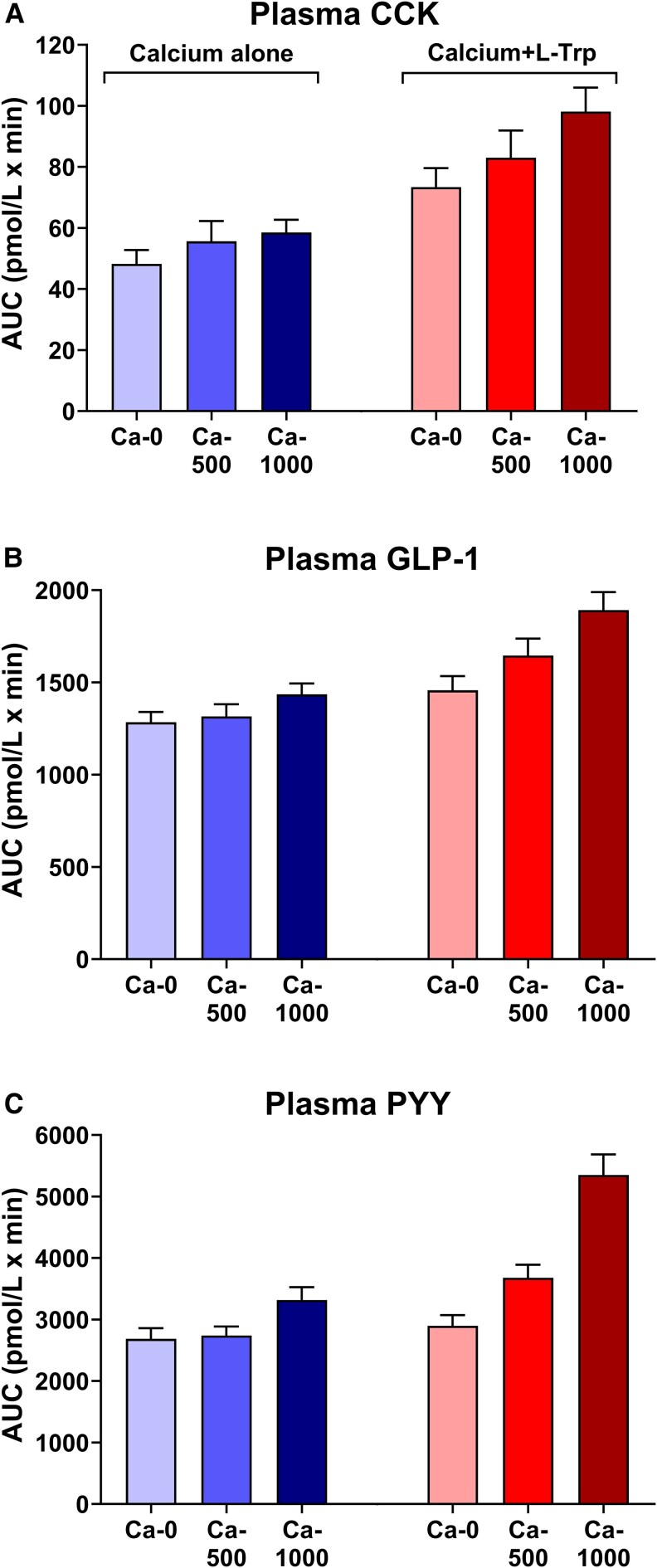
Dose-related effects of intraduodenal administration of 0 mg (‘Ca-0’), 500 mg (“Ca-500”) or 1000 mg (“Ca-1000”) Ca^2+^, as a solution of CaCl_2_, alone (left columns in shades of blue) and in combination with L-tryptophan (L-Trp, right columns in shades of red) on areas under the curve (AUCs) of plasma concentrations of cholecystokinin (CCK) (A), glucagon-like peptide-1 (GLP-1) (B) and peptide YY (PYY) (C) in healthy lean males. Ca-1000 alone stimulated plasma GLP-1 and PYY, but not CCK. Both Ca-500 and Ca-1000 enhanced the effects of L-Trp, in a load of 0.1 kcal/min, to increase plasma CCK, GLP-1, and PYY. Data are expressed as means ± SEMs; n = 15. Figure is based on data from Anjom-Shoae et al (36).

To better characterize the potential effects of Ca^2+^ per se on GI hormone levels, 2 recent well-controlled studies in healthy lean males (36) and individuals with obesity (37), performed by our group, isolated Ca^2+^ from other nutrients by administering Ca^2+^, as a solution of CaCl_2_, directly into the duodenum. In both these studies, Ca^2+^, in a dose of 1000 mg, markedly increased plasma GLP-1 and PYY **(**Fig. 4**)**, but not CCK or GIP, levels. As outlined above, whether these effects are mediated by the CaSR, and whether these effects of Ca^2+^ on GI hormone are associated with a reduction in energy intake and/or lowering of postprandial plasma glucose levels, now warrants investigation, for example, in the absence or presence of calcilytics.

## Effects of L-Trp and L-Phe on GI Hormones, Energy Intake, and Blood Glucose

Various in vitro and in vivo studies have reported that the CaSR mediates L-Trp- or L-Phe-induced stimulation of GI hormone secretion (Table 1) (21-23, 26, 27). Consistent with these findings, human and mouse studies have demonstrated that L-Trp (55-61) and L-Phe (48-51) increase plasma concentrations of GI hormones, suppress energy intake, and reduce the rise in plasma glucose in response to a meal.

### Preclinical Outcomes

As early as ∼1995, L-Phe-induced stimulation of CCK release from STC-1 cells was reported to be dependent on a Ca^2+^-dependent process (141). More recent in vitro studies have also demonstrated that the CaSR mediates L-Trp- or L-Phe-stimulated release of a number of GI hormones (21-23, 26, 27). For example, perfusion of pig gastric tissue with L-Trp or L-Phe increased expression of the CaSR (21), and the effects of L-Trp or L-Phe to stimulate gastrin secretion were enhanced by the CaSR activator cinacalcet and abolished by the CaSR inhibitor, NPS-2143 (21). The effects of L-Trp or L-Phe to stimulate CCK release from isolated mouse or pig small intestine were also inhibited in the presence of the CaSR inhibitors Calhex231 (by ∼55% for L-Trp and ∼25% for L-Phe) (23) or NPS-2143 (L-Trp- or L-Phe-induced peak plasma CCK was reduced from ∼0.20-0.25 ng/mL to ∼0.08-0.18 ng/mL) (25, 26). Furthermore, L-Phe-stimulated CCK secretion from the rat small intestine was completely blocked in the presence of NPS-2143 (22). Similar effects were also observed for other GI hormones. Thus, in isolated loops of rat intestine, the stimulatory effects of L-Trp or L-Phe on GIP, GLP-1, and PYY secretion were blocked by Calhex231 and augmented by the CaSR activator NPS-R568 (27). In porcine duodenal tissue, L-Phe also stimulated GIP release, and the effect was enhanced by cinacalcet, and attenuated by NPS-2143 (peak plasma GIP was reduced from ∼130 ng/mL to ∼70 ng/mL) (26).

There has been less attention to the potential role of the CaSR in mediating L-Trp- (30) or L-Phe-induced (20, 28) stimulation of GI hormone secretion in vivo, and whether these effects impact energy intake or blood glucose levels. In rats, for example, intraduodenal administration of L-Trp increased plasma GLP-1, but not PYY, levels (30), an effect associated with a modest reduction in the fasting blood glucose concentration (30). Both the elevation in plasma GLP-1 levels and reduction in fasting blood glucose were blocked by co-administration of the CaSR inhibitor NPS-2143 (30). Furthermore, in a study on transgenic mice, oral administration of L-Phe stimulated gastrin and gastric acid secretion in wild-type but not in CaSR-null mice (20), and the effect in wild-type mice was completely blocked by NPS-2143. In another study in rats and mice, administration of NPS-2143 blocked L-Phe-induced GLP-1 secretion and reversed L-Phe-induced reductions in food intake (28).

### Clinical Outcomes

Findings in human studies are consistent with the preclinical data with respect to the effects of aromatic amino acids on GI hormones, but studies have not investigated how CaSR agonists/inhibitors modulate such effects; accordingly, a clear mechanistic role of the CaSR has not been established. In several studies, performed by us and other groups, L-Trp (55-62, 142) or L-Phe (48-53), administered mainly orally in capsules or directly into the small intestine or stomach (ie, via nasoduodenal or orogastric catheter), or IV, potently increased plasma GI hormone levels (Table 3). For example, IV infusion of L-Trp (in graded doses of 1.25-5 mmol/h) or L-Phe (in doses of 3.1-12.5 mmol/h), over 4 hours, increased plasma gastrin levels and also stimulated gastric acid secretion (143), and oral or rapid intragastric administration of L-Trp or L-Phe stimulated gastrin secretion in healthy individuals (144, 145). Moreover, oral consumption of 1.5 g L-Trp markedly elevated plasma CCK levels and modestly increased plasma GLP-1 in both healthy lean participants and individuals with obesity (59). In a further study, by our group, intraduodenal infusion of L-Trp for 90 minutes, in a caloric load of 0.15 kcal/min (providing 3.3 g over 90 minutes), but not 0.075 kcal/min (1.65 g over 90 minutes), increased plasma concentrations of CCK, GLP-1, and PYY (56). Similar effects on plasma CCK levels have been observed for L-Phe, when administered intraduodenally (in doses of 3.3 and 5 g) (48, 52), or intragastrically (53) or orally (49) (in a dose of 10 g). In contrast, L-Phe, administered alone either as an oral dose of 10 g (49), or as an intragastric dose of 5 or 10 g (53), did not significantly increase plasma GLP-1 levels.

**Table 3.** Effects of L-tryptophan or L-phenylalanine on plasma concentrations of gut hormones, energy intake, and plasma glucose in humans

Study population	N	Age (y)	BMI (kg/m^2^)	Dose (g)	Type of intervention	Duration	Outcomes			Ref
							Gut hormones	Energy intake (kcal)	Plasma glucose (mmol/L)	
**L-Trp**										
Healthy lean	24 (M/F)	21	24 ± 2	1.4	Oral	180	↑ GLP-1	+11	−0.3	(55)
Healthy lean	10 (M)	26 ± 8	22 ± 2	3.3	ID infusion	90	↑ CCK	−219	NR	(56)
							↑ GLP-1			
							↑ PYY			
Healthy lean Obesity	10 (M/F)	24 ± 1	21 ± 1	1.5	IG bolus	120	↑ CCK	NR	<−0.5	(59)
							↑ GLP-1			
	10 (M/F)	27 ± 8	40 ± 4	1.5	IG bolus		↑ CCK			
							↑ GLP-1			
Healthy lean Obesity	16 (M)	31 ± 3	22 ± 1	3	IG bolus	90	↔ CCK	98	−0.4	(62)
	16 (M)	32 ± 3	33 ± 1	3			↔ CCK	−48	∼−1.0	
Healthy lean	16 (M)	24 ± 1	22.9 ± 2	2.2	ID infusion	90	↑ CCK	+37	NR	(57)
							↔ GLP-1			
Healthy lean Obesity	12 (M)	30 ± 3	23 ± 1	3	IG bolus	150	↑ CCK	−217	NR	(61)
	13(M)	31 ± 3	33 ± 1	3	IG bolus		↑ CCK	−237		
Healthy lean	12 (M)	28 ± 7	23.8 ± 2	1.1	ID infusion	45	↔ CCK	NR	<−0.5	(58)
							↔ GLP-1			
Healthy lean	15 (M)	26 ± 7	22 ± 2	1.8 L-Trp + 500 mg Ca^2+^	ID infusion	150	↔ gastrin	−147	NR	(36)
							↑ CCK			
							↔ GIP			
							↑ GLP-1			
							↑ PYY			
				1.8 g L-Trp + 1000 mg Ca^2+^			↔ gastrin	−186		
							↑ CCK			
							↓GIP			
							↑ GLP-1			
							↑ PYY			
Obesity	15 (M)	27 ± 8	30 ± 2	1.8 L-Trp + 500 mg Ca^2+^	ID infusion	150	↔ gastrin	−70	NR	(37)
							↑ CCK			
							↔ GIP			
							↑ GLP-1			
							↑ PYY			
				1.8 g L-Trp + 1000 mg Ca^2+^			↔ gastrin	−172		
							↑ CCK			
							↔ GIP			
							↑ GLP-1			
							↑ PYY			
**L-Phe**										
Healthy lean	12 (M)	19-34	<25	5	ID infusion	90	↑ CCK	NR	NR	(48)
Healthy lean	5 (M)	25 ± 1	21 ± 2	20	ID infusion	40	↑ CCK	NR	↔	(51)
Healthy lean	6 (M/F)	30 ± 1	20-24	10	Oral	40	↑ CCK	−498	NR	(49)
Healthy lean	16 (M)	23 ± 2	25 ± 1	10	ID infusion	60	↑ CCK	NR	NR	(52)
Healthy lean	10 (M)	23 ± 1	22 ± 1	10	IG bolus	90	↑ CCK	−184	<−1.0	(53)
							↔ GLP-1			
							↑ PYY			
Healthy lean	11 (M/F)	30 ± 1	22 ± 2	10	Oral	150	↑ GIP	−89	∼−1.0	(50)
							↔ GLP-1			
							↑ PYY			

Abbreviations: BMI, body mass index; Ca^2+^, calcium; CCK, cholecystokinin; F, female; GIP, glucose-dependent insulinotropic polypeptide; GLP-1, glucagon-like peptide-1; ID, intraduodenal; IG, intragastric; L-Phe, L-phenylalanine; L-Trp, L-tryptophan; M, male; NR, not reported; PYY, peptide YY; y, years.

L-Trp (56, 59, 61, 62, 142) or L-Phe (49, 50, 53, 54) have also been found to reduce energy intake. For example, we and others have reported that oral administration of L-Trp, in doses of 2 to 3 g, or intragastric administration in a dose of 3.3 g, reduced energy intake substantially in both healthy lean males (61, 146) and in individuals with obesity (61, 147), by up to 200 kcal. Similarly, oral administration of L-Phe (in a dose of 10 g) decreased energy intake in one study in healthy volunteers by 498 kcal (49), and in another study, intragastric or oral administration of L-Phe, in a dose of 10 g, reduced energy intake by 200 (53) or 89 (50) kcal, respectively.

In contrast, the glucose-lowering effects of L-Trp or L-Phe appear to be more modest (50, 53, 54, 62, 142). For example, intragastric administration of 3 g L-Trp as an aqueous solution, 30 minutes before a carbohydrate-containing drink, delayed the rise in plasma glucose in individuals with type 2 diabetes (142), and modestly attenuated the glycemic response in those with obesity by ∼1 mmol/L (62).

## Interactions of Ca^2+^ With L-Trp and L-Phe on GI Hormone Secretion

A number of preclinical studies have investigated whether the stimulatory effects of L-Trp or L-Phe on GI hormone release are dependent on, or positively modulated by, extracellular Ca^2+^ concentration (22, 24-26). Two recent clinical studies have also provided the first evidence that Ca^2+^ enhances the stimulatory effects of L-Trp on GI hormone release in humans (36, 37). The following sections review evidence on the interaction between the effects of Ca^2+^ and the aromatic AAs, L-Trp, and L-Phe, on plasma levels of GI hormones, from both preclinical models and humans. It is important, however, to reiterate that such observations do not allow conclusions as to a direct role for the CaSR.

### Preclinical Outcomes

The stimulatory effects of L-Trp or L-Phe on gastrin secretion from pig intestinal tissue were abolished in the absence of extracellular Ca^2+^ (21). On the other hand, L-Trp- and L-Phe-stimulated CCK secretion from isolated mouse or porcine small intestine was enhanced by increases in extracellular Ca^2+^ (22, 24-26), and co-perfusion of porcine duodenal tissue with Ca^2+^ enhanced the stimulatory effects of L-Trp or L-Phe on GIP secretion (25, 26). In isolated loops of rat intestine, luminal perfusion of AAs, including L-Phe or L-Trp, in the presence of a physiologically relevant Ca^2+^ concentration (1.25 mM), stimulated the secretion of GIP, GLP-1, and PYY, and these effects were blocked by the CaSR inhibitor Calhex231 (27). In addition, extracellular Ca^2+^ markedly enhanced the stimulatory effect of L-Phe in a concentration-dependent manner between 0.3 and 10 mmol/L (27), and the effect of L-Phe was abolished in the absence of extracellular Ca^2+^ (27). Taken together, these observations demonstrate that extracellular Ca^2+^ is required for, and positively modulates, the stimulatory effects of AAs, as previously reported for the CaSR (17, 19). It is, accordingly, possible that reports in which AAs have been observed to stimulate the secretion of some GI hormones, but not others, reflect differences in the Ca^2+^ concentration thresholds required for the effects. Consistent with this notion, in the perfused rat intestine model described above, the extracellular Ca^2+^ threshold concentrations for the stimulatory effect of L-Phe on different GI hormones were around 1.0mM for GIP, 0.3mM for GLP-1, and 0.1mM for PYY (27). Differences of this magnitude might reflect variations in the levels of CaSR expression and/or in the signaling pathways downstream of the CaSR in hormone-secreting cells. These observations may also explain why the administration of AAs has been less effective with respect to GIP than GLP-1 and PYY in some models, both preclinical and clinical, dependent on the prevailing intestinal luminal Ca^2+^ concentration.

### Clinical Outcomes

In humans, the impact of Ca^2+^ to enhance the effect of L-Trp to stimulate GI hormone secretion and enhance the suppression of energy intake was investigated in 2 recent studies (36, 37). In these studies, the modest effects of intraduodenal infusion of L-Trp, in a load of 0.1 kcal/min, to increase plasma CCK, GLP-1, and PYY levels were shown to be enhanced by the addition of 500 or 1000 mg Ca^2+^ (as a solution of CaCl_2)_, in both healthy lean participants (36) and those with obesity (37) **(**Fig. 4**)**. These effects were also associated with a substantial suppression of energy intake (36, 37), so that Ca^2+^ enhanced the suppressive effects of L-Trp on energy intake in a Ca^2+^ dose-related manner. In contrast to CCK, GLP-1, and PYY, the stimulation of GIP by L-Trp was reduced by Ca^2+^ (36). This apparent discrepancy is consistent with previous studies in humans, in which GIP secretion exhibited atypical responses to some nutrients, such as fructose (148, 149). Moreover, no effect on gastrin levels was observed, suggesting minimal, if any, interaction of Ca^2+^ or L-Trp with the G cells in the stomach in this study (36, 37). Moreover, as noted above with respect to the Ca^2+^ concentration thresholds for AA effects, it is possible that the Ca^2+^ concentration ranges over which positive interactions occur with AAs also differ between GI hormone-secreting cell types, and that the effects for GIP and gastrin were maximal in the presence of the baseline Ca^2+^ concentration.

Taken together, these findings indicate that Ca^2+^ has the potential to enhance the effect of L-Trp on the release of several GI hormones in humans, most notably CCK, GLP-1, and PYY, but not others (GIP and gastrin being notable exceptions), and that the outcomes are dependent on the Ca^2+^ dose. The findings support preclinical evidence that extracellular Ca^2+^ enhances the effects of L-Trp on these (22-30) and other GI hormones (20, 21). Clinical evidence for any positive effects of L-Phe, as well as the role of the CaSR in the above effects, is awaited with considerable interest.

## Summary and Future Directions

Since it was first cloned, the CaSR has been the subject of intense research, in part, due to its multiple nutrient binding sites. The dimeric structure of the CaSR supports the binding of AAs as well as extracellular Ca^2+^, thereby displaying diverse physiological impacts. Preclinical studies have established that the CaSR mediates AA-induced secretion of several gut hormones, including gastrin, CCK, GLP-1, GIP, and PYY, with the most potent responses observed for aromatic AAs, including L-Trp and L-Phe. Moreover, these effects are dependent on extracellular Ca^2+^, with distinct threshold Ca^2+^ concentrations required for the effects of AAs on different hormones. Of these GI hormones, the secretion of GLP-1 and PYY are particularly sensitive to elevations in the Ca^2+^ and AA concentrations. Recent human data have also provided evidence that administration of Ca^2+^ dose-dependently enhances the effects of L-Trp to increase plasma levels of CCK, GLP-1, and PYY, associated with greater suppression of energy intake. These findings underscore the intriguing possibility that Ca^2+^ and L-Trp (along with other AAs) act synergistically in humans, as well as animal models, to achieve metabolic benefits through enhanced stimulation of GI hormone release.

While the advent and highly successful application of GLP-1 receptor agonists (150) and the dual GIP/GLP-1 receptor agonist, tirzepatide (151), attest to the therapeutic potential of targeting gut hormone pathways, current pharmacological treatments for the management of obesity and type 2 diabetes are not without limitations, including variable efficacy and high cost, and they are frequently associated with adverse effects, particularly GI symptoms (152), resulting in high discontinuation rates in clinical practice (153). Furthermore, cessation of successful obesity therapy is usually associated with rebound weight gain (154). Thus, targeted nutritional interventions designed to accentuate endogenous gut hormone release remain attractive and have several notable advantages. First, nutritional interventions are expected to concurrently stimulate the release of multiple hormones, including CCK, GLP-1, and PYY, thereby amplifying their collective impacts on appetite suppression and glycemic control (8), while minimizing nonphysiological adverse effects. Secondly, while GLP-1 receptor agonists act primarily via central mechanisms (155), nutrient-induced endogenous GLP-1 release is expected to engage beneficial local GI mechanisms, including locally via receptors on vagal afferent nerve endings, and indirectly through slowing of gastric emptying (156, 157). Finally, while nutrients evoke physiological patterns of hormone secretion and, in turn, receptor activation, sustained activation of GLP-1 receptors via drug agonists may promote receptor downregulation and thereby reduce efficacy in the longer term, so-called “tachyphylaxis.” Accordingly, physiologically informed, well-tolerated, and cost-effective nutritional strategies, either as standalone therapies or as adjuncts to pharmacological treatments, are required for the management of obesity and type 2 diabetes.

Future research should explore the dose-response relationship and longer-term effects of combined administration of Ca^2+^ with L-Trp, L-Phe, and other effective AAs, on energy intake, and postprandial glycemia, as well as body weight, in both healthy people and in metabolically impaired populations. Furthermore, more research is required in humans to directly investigate the role of the CaSR in mediating these effects. This includes evaluating whether nutrient-induced hormone release is CaSR-dependent in vivo, using CaSR antagonists in individuals expressing the wild-type CaSR and individuals with selected mutations of the AA binding site. Additionally, the development and testing of selective CaSR agonists (eg, recently described very high-potency calcimimetics (158)) may represent a novel therapeutic avenue for enhancing endogenous gut hormone secretion without systemic side effects.

## Abbreviations

AA: L-amino acid
CaSR: calcium-sensing receptor
CCK: cholecystokinin
CRD: cysteine-rich domain
GI: gastrointestinal
GIP: glucose-dependent insulinotropic polypeptide
GLP-1: glucagon-like peptide-1
GPR: G protein-coupled receptor
IV: intravenous
L-Phe: L-phenylalanine
L-Trp: L-tryptophan
PTH: parathyroid hormone
PYY: peptide YY
VFT: venus fly trap

## References

[1] Gribble FM, Reimann F. Enteroendocrine cells: chemosensors in the intestinal epithelium. Annu Rev Physiol. 2016;78(1):277‐299. 26442437 10.1146/annurev-physiol-021115-105439

[2] Steensels S, Depoortere I. Chemoreceptors in the gut. Annu Rev Physiol. 2018;80(1):117‐141. 29029594 10.1146/annurev-physiol-021317-121332

[3] Davison A, Reimann F, Gribble FM. Molecular mechanisms of stimulus detection and secretion in enteroendocrine cells. Curr Opin Neurobiol. 2025;92:103045. 40378579 10.1016/j.conb.2025.103045

[4] Gribble FM, Reimann F, Roberts GP. Gastrointestinal hormones. In: Said HM, ed. Physiology of the Gastrointestinal Tract. 6th ed. Academic Press; 2018:31‐70.

[5] Fothergill LJ, Furness JB. Diversity of enteroendocrine cells investigated at cellular and subcellular levels: the need for a new classification scheme. Histochem Cell Biol. 2018;150(6):693‐702. 30357510 10.1007/s00418-018-1746-x PMC6447040

[6] Bany Bakar R, Reimann F, Gribble FM. The intestine as an endocrine organ and the role of gut hormones in metabolic regulation. Nat Rev Gastroenterol Hepatol. 2023;20(12):784‐796. 37626258 10.1038/s41575-023-00830-y

[7] Anjom-Shoae J, Feinle-Bisset C, Horowitz M. Impacts of dietary animal and plant protein on weight and glycemic control in health, obesity and type 2 diabetes: friend or foe? Front Endocrinol (Lausanne). 2024;15:1412182. 39145315 10.3389/fendo.2024.1412182 PMC11321983

[8] Steinert RE, Feinle-Bisset C, Asarian L, Horowitz M, Beglinger C, Geary N. Ghrelin, CCK, GLP-1, and PYY (3–36): secretory controls and physiological roles in eating and glycemia in health, obesity, and after RYGB. Physiol Rev. 2017;97(1):411‐463. 28003328 10.1152/physrev.00031.2014 PMC6151490

[9] Brown EM, Gamba G, Riccardi D, et al Cloning and characterization of an extracellular Ca(^2+^)-sensing receptor from bovine parathyroid. Nature. 1993;366(6455):575‐580. 8255296 10.1038/366575a0

[10] Brown EM, MacLeod RJ. Extracellular calcium sensing and extracellular calcium signaling. Physiol Rev. 2001;81(1):239‐297. 11152759 10.1152/physrev.2001.81.1.239

[11] Chanpaisaeng K, Teerapornpuntakit J, Wongdee K, Charoenphandhu N. Emerging roles of calcium-sensing receptor in the local regulation of intestinal transport of ions and calcium. Am J Physiol Cell Physiol. 2021;320(3):C270‐C278. 33356945 10.1152/ajpcell.00485.2020

[12] Diao J, DeBono A, Josephs TM, et al Therapeutic opportunities of targeting allosteric binding sites on the calcium-sensing receptor. ACS Pharmacol Transl Sci. 2021;4(2):666‐679. 33860192 10.1021/acsptsci.1c00046 PMC8033781

[13] Díaz-Soto G, Rocher A, García-Rodríguez C, Núñez L, Villalobos C. The calcium-sensing receptor in health and disease. Int Rev Cell Mol Biol. 2016;327:321‐369. 27692178 10.1016/bs.ircmb.2016.05.004

[14] Hannan FM, Thakker RV. Calcium-sensing receptor (CaSR) mutations and disorders of calcium, electrolyte and water metabolism. Best Pract Res Clin Endocrinol Metab. 2013;27(3):359‐371. 23856265 10.1016/j.beem.2013.04.007

[15] Nemeth EF, Goodman WG. Calcimimetic and calcilytic drugs: feats, flops, and futures. Calcif Tissue Int. 2016;98(4):341‐358. 26319799 10.1007/s00223-015-0052-z

[16] Pearce SH, Bai M, Quinn SJ, Kifor O, Brown EM, Thakker RV. Functional characterization of calcium-sensing receptor mutations expressed in human embryonic kidney cells. J Clin Invest. 1996;98(8):1860‐1866. 8878438 10.1172/JCI118987 PMC507626

[17] Conigrave AD, Quinn SJ, Brown EM. L-amino acid sensing by the extracellular Ca2+-sensing receptor. Proc Natl Acad Sci U S A. 2000;97(9):4814‐4819. 10781086 10.1073/pnas.97.9.4814 PMC18315

[18] Conigrave AD, Quinn SJ, Brown EM. Cooperative multi-modal sensing and therapeutic implications of the extracellular Ca^2+^ sensing receptor. Trends Pharmacol Sci. 2000;21(10):401‐407. 11050321 10.1016/s0165-6147(00)01546-7

[19] Conigrave AD, Brown EM. Taste receptors in the gastrointestinal tract. II. L-amino acid sensing by calcium-sensing receptors: implications for GI physiology. Am J Physiol Gastrointest Liver Physiol. 2006;291(5):G753‐G761. 17030896 10.1152/ajpgi.00189.2006

[20] Feng J, Petersen CD, Coy DH, et al Calcium-sensing receptor is a physiologic multimodal chemosensor regulating gastric G-cell growth and gastrin secretion. Proc Natl Acad Sci U S A. 2010;107(41):17791‐17796. 20876097 10.1073/pnas.1009078107 PMC2955134

[21] Xian Y, Zhao X, Wang C, et al Phenylalanine and tryptophan stimulate gastrin and somatostatin secretion and H^+^-K^+^-ATPase activity in pigs through calcium-sensing receptor. Gen Comp Endocrinol. 2018;267:1‐8. 29782837 10.1016/j.ygcen.2018.05.022

[22] Hira T, Nakajima S, Eto Y, Hara H. Calcium-sensing receptor mediates phenylalanine-induced cholecystokinin secretion in enteroendocrine STC-1 cells. FEBS J. 2008;275(18):4620‐4626. 18691347 10.1111/j.1742-4658.2008.06604.x

[23] Wang Y, Chandra R, Samsa LA, et al Amino acids stimulate cholecystokinin release through the Ca2+-sensing receptor. Am J Physiol Gastrointest Liver Physiol. 2011;300(4):G528‐G537. 21183662 10.1152/ajpgi.00387.2010 PMC3074989

[24] Liou AP, Sei Y, Zhao X, et al The extracellular calcium-sensing receptor is required for cholecystokinin secretion in response to L-phenylalanine in acutely isolated intestinal I cells. Am J Physiol Gastrointest Liver Physiol. 2011;300(4):G538‐G546. 21252045 10.1152/ajpgi.00342.2010 PMC3074990

[25] Zhao X, Xian Y, Wang C, et al Calcium-sensing receptor-mediated L-tryptophan-induced secretion of cholecystokinin and glucose-dependent insulinotropic peptide in swine duodenum. J Vet Sci. 2018;19(2):179‐187. 29284209 10.4142/jvs.2018.19.2.179 PMC5879066

[26] Feng J, Kang C, Wang C, Ding L, Zhu W, Hang S. L-phenylalanine increased gut hormone secretion through calcium-sensing receptor in the porcine duodenum. Animals (Basel). 2019;9(8):476. 31344840 10.3390/ani9080476 PMC6719913

[27] Mace OJ, Schindler M, Patel S. The regulation of K-and L-cell activity by GLUT2 and the calcium-sensing receptor CasR in rat small intestine. J Physiol. 2012;590(12):2917‐2936. 22495587 10.1113/jphysiol.2011.223800 PMC3448156

[28] Alamshah A, Spreckley E, Norton M, et al L-phenylalanine modulates gut hormone release and glucose tolerance, and suppresses food intake through the calcium-sensing receptor in rodents. Int J Obes (Lond). 2017;41(11):1693‐1701. 28792489 10.1038/ijo.2017.164 PMC5678004

[29] Wang H, Murthy KS, Grider JR. Expression patterns of L-amino acid receptors in the murine STC-1 enteroendocrine cell line. Cell Tissue Res. 2019;378(3):471‐483. 31410629 10.1007/s00441-019-03074-y PMC6872933

[30] Acar I, Cetinkaya A, Lay I, Ileri-Gurel E. The role of calcium sensing receptors in GLP-1 and PYY secretion after acute intraduodenal administration of L-tryptophan in rats. Nutr Neurosci. 2020;23(6):481‐489. 30222528 10.1080/1028415X.2018.1521906

[31] Igarashi A, Ogasawara S, Takagi R, et al Acute oral calcium suppresses food intake through enhanced peptide-YY secretion mediated by the calcium-sensing receptor in rats. J Nutr. 2021;151(5):1320‐1328. 33693689 10.1093/jn/nxab013

[32] Li RJW, Barros DR, Kuah R, et al Small intestinal CaSR-dependent and CaSR-independent protein sensing regulates feeding and glucose tolerance in rats. Nat Metab. 2024;6(1):39‐49. 38167726 10.1038/s42255-023-00942-4

[33] Rybczyńska A, Marchwińska A, Dyś A, Boblewski K, Lehmann A, Lewko B. Activity of the calcium-sensing receptor influences blood glucose and insulin levels in rats. Pharmacol Rep. 2017;69(4):709‐713. 28551530 10.1016/j.pharep.2017.01.034

[34] Beumer J, Geurts MH, Geurts V, et al Description and functional validation of human enteroendocrine cell sensors. Science. 2024;386(6719):341‐348. 39418382 10.1126/science.adl1460 PMC7616728

[35] Guccio N, Alcaino C, Miedzybrodzka EL, et al Molecular mechanisms underlying glucose-dependent insulinotropic polypeptide secretion in human duodenal organoids. Diabetologia. 2025;68(1):217‐230. 39441374 10.1007/s00125-024-06293-3 PMC11663192

[36] Anjom-Shoae J, Fitzgerald PC, Horowitz M, et al Intraduodenal calcium enhances the effects of L-tryptophan to stimulate gut hormone secretion and suppress energy intake in healthy males: a randomized, crossover, clinical trial. Am J Clin Nutr. 2024;120(3):528‐539. 38996913 10.1016/j.ajcnut.2024.07.006

[37] Anjom-Shoae J, Fitzgerald PCE, Horowitz M, et al Dose-related effects of calcium to enhance the effects of L-tryptophan on gut hormones and energy intake in obesity. J Clin Endocrinol Metab. 2025;110(9):e2929‐e2938. 39785828 10.1210/clinem/dgaf008 PMC12342367

[38] Lorenzen JK, Nielsen S, Holst JJ, Tetens I, Rehfeld JF, Astrup A. Effect of dairy calcium or supplementary calcium intake on postprandial fat metabolism, appetite, and subsequent energy intake. Am J Clin Nutr. 2007;85(3):678‐687. 17344487 10.1093/ajcn/85.3.678

[39] Gonzalez JT, Stevenson EJ. Calcium co-ingestion augments postprandial glucose-dependent insulinotropic peptide 1–42, glucagon-like peptide-1 and insulin concentrations in humans. Eur J Nutr. 2014;53(2):375‐385. 23689561 10.1007/s00394-013-0532-8

[40] Chen Y-C, Smith HA, Hengist A, et al Co-ingestion of whey protein hydrolysate with milk minerals rich in calcium potently stimulates glucagon-like peptide-1 secretion: an RCT in healthy adults. Eur J Nutr. 2020;59(6):2449‐2462. 31531707 10.1007/s00394-019-02092-4 PMC7413905

[41] Jones KW, Eller LK, Parnell JA, Doyle-Baker PK, Edwards AL, Reimer RA. Effect of a dairy-and calcium-rich diet on weight loss and appetite during energy restriction in overweight and obese adults: a randomized trial. Eur J Clin Nutr. 2013;67(4):371‐376. 23462943 10.1038/ejcn.2013.52 PMC3948984

[42] Trautvetter U, Jahreis G. Effect of supplementary calcium phosphate on plasma gastrointestinal hormones in a double-blind, placebo-controlled, cross-over human study. Br J Nutr. 2014;111(2):287‐293. 23871132 10.1017/S0007114513002341

[43] Gonzalez JT, Green BP, Campbell M, Rumbold P, Stevenson E. The influence of calcium supplementation on substrate metabolism during exercise in humans: a randomized controlled trial. Eur J Clin Nutr. 2014;68(6):712‐718. 24642785 10.1038/ejcn.2014.41

[44] Gonzalez JT, Green BP, Brown MA, Rumbold PL, Turner LA, Stevenson EJ. Calcium ingestion suppresses appetite and produces acute overcompensation of energy intake independent of protein in healthy adults. J Nutr. 2015;145(3):476‐482. 25733462 10.3945/jn.114.205708

[45] Wang C, Kang C, Xian Y, et al Sensing of L-arginine by gut-expressed calcium sensing receptor stimulates gut satiety hormones cholecystokinin and glucose-dependent insulinotropic peptide secretion in pig model. J Food Sci. 2018;83(9):2394‐2401. 30088839 10.1111/1750-3841.14297

[46] Modvig IM, Kuhre RE, Holst JJ. Peptone-mediated glucagon-like peptide-1 secretion depends on intestinal absorption and activation of basolaterally located calcium-sensing receptors. Physiol Rep. 2019;7(8):e14056. 31020803 10.14814/phy2.14056 PMC6482282

[47] Conigrave AD, Mun HC, Delbridge L, Quinn SJ, Wilkinson M, Brown EM. L-amino acids regulate parathyroid hormone secretion. J Biol Chem. 2004;279(37):38151‐38159. 15234970 10.1074/jbc.M406373200

[48] Owyang C, Louie DS, Tatum D. Feedback regulation of pancreatic enzyme secretion. Suppression of cholecystokinin release by trypsin. J Clin Invest. 1986;77(6):2042‐2047. 3711342 10.1172/JCI112534 PMC370566

[49] Ballinger A, Clark M. L-phenylalanine releases cholecystokinin (CCK) and is associated with reduced food intake in humans: evidence for a physiological role of CCK in control of eating. Metabolism. 1994;43(6):735‐738. 8201963 10.1016/0026-0495(94)90123-6

[50] Amin A, Frampton J, Liu Z, et al Differential effects of L-and D-phenylalanine on pancreatic and gastrointestinal hormone release in humans: a randomized crossover study. Diabetes Obes Metab. 2021;23(1):147‐157. 32991046 10.1111/dom.14204

[51] Reimers J, Nauck M, Creutzfeldt W, et al Lack of insulinotropic effect of endogenous and exogenous cholecystokinin in man. Diabetologia. 1988;31(5):271‐280. 3294066 10.1007/BF00277407

[52] Steinert RE, Landrock MF, Horowitz M, Feinle-Bisset C. Effects of intraduodenal infusions of L-phenylalanine and L-glutamine on antropyloroduodenal motility and plasma cholecystokinin in healthy men. J Neurogastroenterol Motil. 2015;21(3):404‐413. 26130636 10.5056/jnm14143 PMC4496893

[53] Fitzgerald PCE, Manoliu B, Herbillon B, Steinert RE, Horowitz M, Feinle-Bisset C. Effects of L-phenylalanine on energy intake and glycaemia—impacts on appetite perceptions, gastrointestinal hormones and gastric emptying in healthy males. Nutrients. 2020;12(6):1788. 32560181 10.3390/nu12061788 PMC7353198

[54] Nuttall FQ, Schweim K, Gannon M. Effect of orally administered phenylalanine with and without glucose on insulin, glucagon and glucose concentrations. Horm Metab Res. 2006;38(8):518‐523. 16941278 10.1055/s-2006-949523

[55] Nieuwenhuizen AG, Hochstenbach-Waelen A, Veldhorst MA, et al Acute effects of breakfasts containing α-lactalbumin, or gelatin with or without added tryptophan, on hunger, ‘satiety’hormones and amino acid profiles. Br J Nutr. 2009;101(12):1859‐1866. 19017422 10.1017/S0007114508131774

[56] Steinert RE, Luscombe-Marsh ND, Little TJ, et al Effects of intraduodenal infusion of L-tryptophan on ad libitum eating, antropyloroduodenal motility, glycemia, insulinemia, and gut peptide secretion in healthy men. J Clin Endocrinol Metab. 2014;99(9):3275‐3284. 24926954 10.1210/jc.2014-1943

[57] McVeay C, Fitzgerald PC, Ullrich SS, Steinert RE, Horowitz M, Feinle-Bisset C. Effects of intraduodenal administration of lauric acid and L-tryptophan, alone and combined, on gut hormones, pyloric pressures, and energy intake in healthy men. Am J Clin Nutr. 2019;109(5):1335‐1343. 31051504 10.1093/ajcn/nqz020

[58] Hajishafiee M, McVeay C, Lange K, Rehfeld J, Horowitz M, Feinle-Bisset C. Effects of intraduodenal infusion of lauric acid and L-tryptophan, alone and combined, on glucoregulatory hormones, gastric emptying and glycaemia in healthy men. Metabolism. 2022;129:155140. 35065080 10.1016/j.metabol.2022.155140

[59] Meyer-Gerspach AC, Häfliger S, Meili J, et al Effect of L-tryptophan and L-leucine on gut hormone secretion, appetite feelings and gastric emptying rates in lean and non-diabetic obese participants: a randomized, double-blind, parallel-group trial. PLoS One. 2016;11(11):e0166758. 27875537 10.1371/journal.pone.0166758 PMC5119776

[60] Hajishafiee M, Ullrich SS, Steinert RE, et al Effects of intragastric tryptophan on acute changes in the plasma tryptophan/large neutral amino acids ratio and relationship with subsequent energy intake in lean and obese men. Food Funct. 2020;11(8):7095‐7103. 32729586 10.1039/d0fo00773k

[61] Hajishafiee M, Ullrich SS, Fitzgerald PC, et al Suppression of energy intake by intragastric l-tryptophan in lean and obese men: relations with appetite perceptions and circulating cholecystokinin and tryptophan. J Nutr. 2021;151(10):2932‐2941. 34255069 10.1093/jn/nxab218

[62] Ullrich SS, Fitzgerald PC, Giesbertz P, Steinert RE, Horowitz M, Feinle-Bisset C. Effects of intragastric administration of tryptophan on the blood glucose response to a nutrient drink and energy intake, in lean and obese men. Nutrients. 2018;10(4):463. 29642492 10.3390/nu10040463 PMC5946248

[63] Anjom-Shoae J, Hajishafiee M, Fitzgerald PC, et al Acute decrease in the plasma tryptophan-to-large-neutral-amino-acids ratio attenuates the effects of L-tryptophan on gut hormones and energy intake in healthy males: a randomized, cross-over, exploratory trial. Am J Clin Nutr. 2025;121(4):816‐825. 39978467 10.1016/j.ajcnut.2025.02.016

[64] Modvig IM, Kuhre RE, Jepsen SL, et al Amino acids differ in their capacity to stimulate GLP-1 release from the perfused rat small intestine and stimulate secretion by different sensing mechanisms. Am J Physiol Endocrinol Metab. 2021;320(5):E874‐E885. 33645250 10.1152/ajpendo.00026.2021

[65] Riccardi D, Park J, Lee WS, Gamba G, Brown EM, Hebert SC. Cloning and functional expression of a rat kidney extracellular calcium/polyvalent cation-sensing receptor. Proc Natl Acad Sci U S A. 1995;92(1):131‐135. 7816802 10.1073/pnas.92.1.131 PMC42831

[66] Garrett JE, Capuano IV, Hammerland LG, et al Molecular cloning and functional expression of human parathyroid calcium receptor cDNAs. J Biol Chem. 1995;270(21):12919‐12925. 7759551 10.1074/jbc.270.21.12919

[67] Geng Y, Mosyak L, Kurinov I, et al Structural mechanism of ligand activation in human calcium-sensing receptor. Elife. 2016;5:e13662. 27434672 10.7554/eLife.13662 PMC4977154

[68] Gao Y, Robertson MJ, Rahman SN, et al Asymmetric activation of the calcium-sensing receptor homodimer. Nature. 2021;595(7867):455‐459. 34194040 10.1038/s41586-021-03691-0 PMC8826748

[69] Park J, Zuo H, Frangaj A, et al Symmetric activation and modulation of the human calcium-sensing receptor. Proc Natl Acad Sci U S A. 2021;118(51):e2115849118. 34916296 10.1073/pnas.2115849118 PMC8713963

[70] Handlogten ME, Shiraishi N, Awata H, Huang C, Miller RT. Extracellular Ca(^2+^)-sensing receptor is a promiscuous divalent cation sensor that responds to lead. Am J Physiol Renal Physiol. 2000;279(6):F1083‐F1091. 11097627 10.1152/ajprenal.2000.279.6.F1083

[71] Bandyopadhyay S, Tfelt-Hansen J, Chattopadhyay N. Diverse roles of extracellular calcium-sensing receptor in the central nervous system. J Neurosci Res. 2010;88(10):2073‐2082. 20336672 10.1002/jnr.22391

[72] Wang M, Yao Y, Kuang D, Hampson DR. Activation of family C G-protein-coupled receptors by the tripeptide glutathione. J Biol Chem. 2006;281(13):8864‐8870. 16455645 10.1074/jbc.M512865200

[73] Yang J, Bai W, Zeng X, Cui C. γ-[Glu](n= 1, 2)-Phe/-Met/-Val stimulates gastrointestinal hormone (CCK and GLP-1) secretion by activating the calcium-sensing receptor. Food Funct. 2019;10(7):4071‐4080. 31225563 10.1039/c9fo00313d

[74] Quinn SJ, Ye CP, Diaz R, et al The Ca^2+^-sensing receptor: a target for polyamines. Am J Physiol. 1997;273(4):C1315‐C1323. 9357776 10.1152/ajpcell.1997.273.4.C1315

[75] Wootten D, Christopoulos A, Marti-Solano M, Babu MM, Sexton PM. Mechanisms of signalling and biased agonism in G protein-coupled receptors. Nat Rev Mol Cell Biol. 2018;19(10):638‐653. 30104700 10.1038/s41580-018-0049-3

[76] Quinn SJ, Kifor O, Trivedi S, Diaz R, Vassilev P, Brown E. Sodium and ionic strength sensing by the calcium receptor. J Biol Chem. 1998;273(31):19579‐19586. 9677383 10.1074/jbc.273.31.19579

[77] Quinn SJ, Bai M, Brown EM. Ph sensing by the calcium-sensing receptor. J Biol Chem. 2004;279(36):37241‐37249. 15201280 10.1074/jbc.M404520200

[78] Conigrave AD, Hampson DR. Broad-spectrum L-amino acid sensing by class 3 G-protein-coupled receptors. Trends Endocrinol Metab. 2006;17(10):398‐407. 17085057 10.1016/j.tem.2006.10.012

[79] Chen RA, Goodman WG. Role of the calcium-sensing receptor in parathyroid gland physiology. Am J Physiol Renal Physiol. 2004;286(6):F1005‐F1011. 15130894 10.1152/ajprenal.00013.2004

[80] Riccardi D, Brown EM. Physiology and pathophysiology of the calcium-sensing receptor in the kidney. Am J Physiol Renal Physiol. 2010;298(3):F485‐F499. 19923405 10.1152/ajprenal.00608.2009 PMC2838589

[81] Garrett JE, Tamir H, Kifor O, et al Calcitonin-secreting cells of the thyroid express an extracellular calcium receptor gene. Endocrinology. 1995;136(11):5202‐5211. 7588259 10.1210/endo.136.11.7588259

[82] Marie PJ . The calcium-sensing receptor in bone cells: a potential therapeutic target in osteoporosis. Bone. 2010;46(3):571‐576. 19660583 10.1016/j.bone.2009.07.082

[83] Alfadda TI, Saleh AMA, Houillier P, Geibel JP. Calcium-sensing receptor 20 years later. Am J Physiol Cell Physiol. 2014;307(3):C221‐C231. 24871857 10.1152/ajpcell.00139.2014 PMC4121584

[84] Saidak Z, Mentaverri R, Brown EM. The role of the calcium-sensing receptor in the development and progression of cancer. Endocr Rev. 2009;30(2):178‐195. 19237714 10.1210/er.2008-0041

[85] Geibel JP, Hebert SC. The functions and roles of the extracellular Ca2+-sensing receptor along the gastrointestinal tract. Annu Rev Physiol. 2009;71(1):205‐217. 19575679 10.1146/annurev.physiol.010908.163128

[86] Brennan SC, Davies TS, Schepelmann M, Riccardi D. Emerging roles of the extracellular calcium-sensing receptor in nutrient sensing: control of taste modulation and intestinal hormone secretion. Br J Nutr. 2014;111(S1):S16‐S22. 24382107 10.1017/S0007114513002250

[87] Ray JM, Squires PE, Curtis SB, Meloche MR, Buchan AM. Expression of the calcium-sensing receptor on human antral gastrin cells in culture. J Clin Invest. 1997;99(10):2328‐2333. 9153273 10.1172/JCI119413 PMC508070

[88] Rácz GZ, Kittel A, Riccardi D, Case RM, Elliott AC, Varga G. Extracellular calcium sensing receptor in human pancreatic cells. Gut. 2002;51(5):705‐711. 12377811 10.1136/gut.51.5.705 PMC1773426

[89] Gong Y, Yang B, Zhang D, et al Hyperaminoacidemia induces pancreatic α cell proliferation via synergism between the mTORC1 and CaSR-Gq signaling pathways. Nat Commun. 2023;14(1):235. 36646689 10.1038/s41467-022-35705-4 PMC9842633

[90] Canaff L, Petit J-L, Kisiel M, Watson PH, Gascon-Barré M, Hendy GN. Extracellular calcium-sensing receptor is expressed in rat hepatocytes. Coupling to intracellular calcium mobilization and stimulation of bile flow. J Biol Chem. 2001;276(6):4070‐4079. 11071898 10.1074/jbc.M009317200

[91] Babinsky VN, Hannan FM, Ramracheya RD, et al Mutant mice with calcium-sensing receptor activation have hyperglycemia that is rectified by calcilytic therapy. Endocrinology. 2017;158(8):2486‐2502. 28575322 10.1210/en.2017-00111 PMC5551547

[92] Rudenko O, Shang J, Munk A, et al The aromatic amino acid sensor GPR142 controls metabolism through balanced regulation of pancreatic and gut hormones. Mol Metab. 2019;19:49‐64. 30472415 10.1016/j.molmet.2018.10.012 PMC6323244

[93] Cheng SX, Lightfoot YL, Yang T, et al Epithelial CaSR deficiency alters intestinal integrity and promotes proinflammatory immune responses. FEBS Lett. 2014;588(22):4158‐4166. 24842610 10.1016/j.febslet.2014.05.007 PMC4234694

[94] Cheng SX, Okuda M, Hall AE, Geibel JP, Hebert SC. Expression of calcium-sensing receptor in rat colonic epithelium: evidence for modulation of fluid secretion. Am J Physiol Gastrointest Liver Physiol. 2002;283(1):G240‐G250. 12065312 10.1152/ajpgi.00500.2001

[95] Iamartino L, Brandi ML. The calcium-sensing receptor in inflammation: recent updates. Front Physiol. 2022;13:1059369. 36467702 10.3389/fphys.2022.1059369 PMC9716066

[96] Cheng I, Qureshi I, Chattopadhyay N, et al Expression of an extracellular calcium-sensing receptor in rat stomach. Gastroenterology. 1999;116(1):118‐126. 9869609 10.1016/s0016-5085(99)70235-0

[97] Igarashi T, Ogata E, Maruyama K, Fukuda T, Azuma J. Effect of calcimimetic agent, KRN568, on gastrin secretion in healthy subjects. Endocr J. 2000;47(5):517‐523. 11200930 10.1507/endocrj.47.517

[98] Ceglia L, Harris SS, Rasmussen HM, Dawson-Hughes B. Activation of the calcium sensing receptor stimulates gastrin and gastric acid secretion in healthy participants. Osteoporos Int. 2009;20(1):71‐78. 18536954 10.1007/s00198-008-0637-8 PMC2716662

[99] Bevilacqua M, Dominguez LJ, Righini V, et al Dissimilar PTH, gastrin, and calcitonin responses to oral calcium and peptones in hypocalciuric hypercalcemia, primary hyperparathyroidism, and normal subjects: a useful tool for differential diagnosis. J Bone Miner Res. 2006;21(3):406‐412. 16491288 10.1359/JBMR.051210

[100] Reeder RH, Brown DD, Wellauer PK, Dawid IB. Patterns of ribosomal DNA spacer lengths are inherited. J Mol Biol. 1976;105(4):507‐516. 972393 10.1016/0022-2836(76)90231-x

[101] Zaniewski M, Jordan PH Jr, Yip B, Thornby JI, Mallette LE. Serum gastrin level is increased by chronic hypercalcemia of parathyroid or nonparathyroid origin. Arch Intern Med. 1986;146(3):478‐482. 2869739

[102] Díez JJ, Miguel JL, Codoceo R, et al Effects of cinacalcet on gastrointestinal hormone release in patients with secondary hyperparathyroidism undergoing dialysis. Nephrol Dial Transplant. 2007;23(4):1387‐1395. 18045826 10.1093/ndt/gfm776

[103] Santos-Hernández M, Reimann F, Gribble FM. Cellular mechanisms of incretin hormone secretion. J Mol Endocrinol. 2024;72(4):e230112. 38240302 10.1530/JME-23-0112 PMC10959011

[104] Gustavsson N, Wang Y, Kang Y, et al Synaptotagmin-7 as a positive regulator of glucose-induced glucagon-like peptide-1 secretion in mice. Diabetologia. 2011;54(7):1824‐1830. 21424898 10.1007/s00125-011-2119-3

[105] Zuo H, Park J, Frangaj A, et al Promiscuous G-protein activation by the calcium-sensing receptor. Nature. 2024;629(8011):481‐488. 38632411 10.1038/s41586-024-07331-1 PMC11844898

[106] Petersen JE, Pedersen MH, Dmytriyeva O, et al Free fatty acid receptor 1 stimulates cAMP production and gut hormone secretion through Gq-mediated activation of adenylate cyclase 2. Mol Metab. 2023;74:101757. 37348738 10.1016/j.molmet.2023.101757 PMC10394103

[107] Barreras RF . Calcium and gastric secretion. Gastroenterology. 1973;64(6):1168‐1184. 4574679

[108] Case RM . Calcium and gastrointestinal secretion. Digestion. 1973;8(3):269‐288. 4354614 10.1159/000197324

[109] Mangel AW, Snow ND, Misukonis M, et al Calcium-dependent regulation of cholecystokinin secretion and potassium currents in STC-1 cells. Am J Physiol. 1993;264(6 Pt 1):G1031‐G1036. 8333529 10.1152/ajpgi.1993.264.6.G1031

[110] Abello J, Ye F, Bosshard A, Bernard C, Cuber JC, Chayvialle JA. Stimulation of glucagon-like peptide-1 secretion by muscarinic agonist in a murine intestinal endocrine cell line. Endocrinology. 1994;134(5):2011‐2017. 8156901 10.1210/endo.134.5.8156901

[111] Bunce KT, Honey AC, Parsons ME. Investigation of the role of extracellular calcium in the control of acid secretion in the isolated whole stomach of the rat. Br J Pharmacol. 1979;67(1):123‐131. 227506 PMC2043604

[112] Black J, Welch L. Are gastrin receptors located on parietal cells [proceedings]? Br J Pharmacol. 1977;59(3):476P. PMC1667977 191130

[113] Main I, Pearce J. Effect of calcium on acid secretion by the isolated rat gastric mucosa [proceedings]. Br J Pharmacol. 1977;61(1):124P. PMC1667669 912176

[114] Basso N, Passaro E Jr. Effect of calcium on pentagastrin-, histamine-, bethanecol-, and insulin-stimulated gastric secretion in the ferret. J Surg Res. 1972;13(1):32‐38. 4626724 10.1016/0022-4804(72)90037-6

[115] Becker HD, Konturek SJ, Reeder DD, Thompson JC. Effect of calcium and calcitonin on gastrin and gastric secretion in cats. Am J Physiol. 1973;225(2):277‐280. 4722388 10.1152/ajplegacy.1973.225.2.277

[116] Harty RF, Maico DG, McGuigan JE. Role of calcium in antral gastrin release. Gastroenterology. 1981;80(3):491‐497. 6256255

[117] Lichtenberger LM, Shaw LS, Bailey RB. Influence of calcium on the release of gastrin from isolated rodent G cells. Proc Soc Exp Biol Med. 1981;166(4):587‐591. 7220550 10.3181/00379727-166-41113

[118] Fiddian-Green RG, Pittenger G, Kothary P, Vinik AI. Role of calcium in the stimulus-secretion coupling of antral gastrin release. Endocrinology. 1983;112(2):753‐760. 6401247 10.1210/endo-112-2-753

[119] Aucouturier S, Cuber JC, Bernard C, Chayvialle JA. Role of calcium in the bombesin-induced intestinal CCK release in rats. Peptides. 1993;14(6):1295‐1297. 8134312 10.1016/0196-9781(93)90189-n

[120] Beinfeld MC, Haun RS, Allard LR, Dixon JE. Regulation of cholecystokinin secretion from a rat medullary thyroid carcinoma cell line: role of calcium, cyclic nucleotides, glucocorticoids, neurotensin, and calcitonin gene-related peptide. Peptides. 1992;13(3):545‐550. 1523166 10.1016/0196-9781(92)90087-j

[121] Bouras EP, Misukonis M, Liddle RA. Role of calcium in monitor peptide-stimulated cholecystokinin release from perifused intestinal cells. Am J Physiol. 1992;262(5 Pt 1):G791‐G796. 1590389 10.1152/ajpgi.1992.262.5.G791

[122] Snow ND, Prpic V, Mangel AW, et al Regulation of cholecystokinin secretion by bombesin in STC-1 cells. Am J Physiol. 1994;267(5 Pt 1):G859‐G865. 7977748 10.1152/ajpgi.1994.267.5.G859

[123] McLaughlin JT, Lomax RB, Hall L, Dockray GJ, Thompson DG, Warhurst G. Fatty acids stimulate cholecystokinin secretion via an acyl chain length-specific, Ca^2+^-dependent mechanism in the enteroendocrine cell line STC-1. J Physiol. 1998;513(Pt 1):11‐18. 9782155 10.1111/j.1469-7793.1998.011by.x PMC2231256

[124] Némoz-Gaillard E, Bernard C, Abello J, Cordier-Bussat M, Chayvialle J-A, Cuber J-C. Regulation of cholecystokinin secretion by peptones and peptidomimetic antibiotics in STC-1 cells. Endocrinology. 1998;139(3):932‐938. 9492022 10.1210/endo.139.3.5802

[125] Nakajima S, Hira T, Yahagi A, et al Unsaturated aldehydes induce CCK secretion via TRPA1 in STC-1 cells. Mol Nutr Food Res. 2014;58(5):1042‐1051. 24357536 10.1002/mnfr.201300412

[126] Wang Y, Prpic V, Green GM, Reeve JR, Liddle RA. Luminal CCK-releasing factor stimulates CCK release from human intestinal endocrine and STC-1 cells. Am J Physiol Gastrointest Liver Physiol. 2002;282(1):G16‐G22. 11751153 10.1152/ajpgi.2002.282.1.G16

[127] Sidhu SS, Thompson DG, Warhurst G, Case RM, Benson RS. Fatty acid-induced cholecystokinin secretion and changes in intracellular Ca2+ in two enteroendocrine cell lines, STC-1 and GLUTag. J Physiol. 2000;528(Pt 1):165‐176. 11018115 10.1111/j.1469-7793.2000.00165.x PMC2270123

[128] Reimann F, Maziarz M, Flock G, Habib A, Drucker DJ, Gribble FM. Characterization and functional role of voltage gated cation conductances in the glucagon-like peptide-1 secreting GLUTag cell line. J Physiol. 2005;563(Pt 1):161‐175. 15611035 10.1113/jphysiol.2004.076414 PMC1665554

[129] Rogers GJ, Tolhurst G, Ramzan A, et al Electrical activity-triggered glucagon-like peptide-1 secretion from primary murine L-cells. J Physiol. 2011;589(Pt 5):1081‐1093. 21224236 10.1113/jphysiol.2010.198069 PMC3060588

[130] Kuhre RE, Frost CR, Svendsen B, Holst JJ. Molecular mechanisms of glucose-stimulated GLP-1 secretion from perfused rat small intestine. Diabetes. 2015;64(2):370‐382. 25157092 10.2337/db14-0807

[131] Buchan AMJ, Squires PE, Ring M, Meloche RM. Mechanism of action of the calcium-sensing receptor in human antral gastrin cells. Gastroenterology. 2001;120(5):1128‐1139. 11266377 10.1053/gast.2001.23246

[132] Remy C, Kirchhoff P, Hafner P, et al Stimulatory pathways of the calcium-sensing receptor on acid secretion in freshly isolated human gastric glands. Cell Physiol Biochem. 2007;19(1-4):33‐42. 17310098 10.1159/000099190

[133] Sun X, Zemel MB. Calcium and dairy products inhibit weight and fat regain during ad libitum consumption following energy restriction in Ap2-agouti transgenic mice. J Nutr. 2004;134(11):3054‐3060. 15514275 10.1093/jn/134.11.3054

[134] Pilvi TK, Harala S, Korpela R, Mervaala EM. Effects of high-calcium diets with different whey proteins on weight loss and weight regain in high-fat-fed C57BL/6J mice. Br J Nutr. 2009;102(3):337‐341. 19622178 10.1017/S0007114508199445

[135] Eller LK, Reimer RA. Attenuation in weight gain with high calcium-and dairy-enriched diets is not associated with taste aversion in rats: a comparison with casein, whey, and soy. J Med Food. 2010;13(5):1182‐1188. 20626247 10.1089/jmf.2009.0223

[136] Dove ER, Hodgson JM, Puddey IB, Beilin LJ, Lee YP, Mori TA. Skim milk compared with a fruit drink acutely reduces appetite and energy intake in overweight men and women. Am J Clin Nutr. 2009;90(1):70‐75. 19474132 10.3945/ajcn.2008.27411

[137] Ping-Delfos WCS, Soares M. Diet induced thermogenesis, fat oxidation and food intake following sequential meals: influence of calcium and vitamin D. Clin Nutr. 2011;30(3):376‐383. 21276644 10.1016/j.clnu.2010.11.006

[138] Kabrnova-Hlavata K, Hainer V, Gojova M, et al Calcium intake and the outcome of short-term weight management. Physiol Res. 2008;57(2):237‐245. 17552880 10.33549/physiolres.931057

[139] Major GC, Alarie FP, Doré J, Tremblay A. Calcium plus vitamin D supplementation and fat mass loss in female very low-calcium consumers: potential link with a calcium-specific appetite control. Br J Nutr. 2009;101(5):659‐663. 19263591 10.1017/s0007114508030808

[140] Gilbert J-A, Joanisse DR, Chaput J-P, et al Milk supplementation facilitates appetite control in obese women during weight loss: a randomised, single-blind, placebo-controlled trial. Br J Nutr. 2011;105(1):133‐143. 21205360 10.1017/S0007114510003119

[141] Mangel AW, Prpic V, Wong H, et al Phenylalanine-stimulated secretion of cholecystokinin is calcium dependent. Am J Physiol. 1995;268(1 Pt 1):G90‐G94. 7840211 10.1152/ajpgi.1995.268.1.G90

[142] Hajishafiee M, Elovaris RA, Jones KL, et al Effects of intragastric administration of L-tryptophan on the glycaemic response to a nutrient drink in men with type 2 diabetes - impacts on gastric emptying, glucoregulatory hormones and glucose absorption. Nutr Diabetes. 2021;11(1):3. 33414406 10.1038/s41387-020-00146-9 PMC7791097

[143] McArthur KE, Isenberg JI, Hogan DL, Dreier SJ. Intravenous infusion of L-isomers of phenylalanine and tryptophan stimulate gastric acid secretion at physiologic plasma concentrations in normal subjects and after parietal cell vagotomy. J Clin Invest. 1983;71(5):1254‐1262. 6853713 10.1172/JCI110875 PMC436986

[144] McCallum RW, Kuljian B, Holloway RH, Walsh JH. Effect of intragastric amino acids on lower esophageal sphincter pressure and serum gastrin in man. Am J Gastroenterol. 1986;81(3):168‐171. 3953532

[145] Taylors IL, Byrne WJ, Christie DL, Ament ME, Walsh JH. Effect of individual L-amino acids on gastric acid secretion and serum gastrin and pancreatic polypeptide release in humans. Gastroenterology. 1982;83(1 Pt 2):273‐278. 6806140

[146] Hrboticky N, Leiter LA, Anderson GH. Effects of L-tryptophan on short term food intake in lean men. Nutr Res. 1985;5(6):595‐607.

[147] Cavaliere H, Medeiros-Neto G. The anorectic effect of increasing doses of L-tryptophan in obese patients. Eat Weight Disord. 1997;2(4):211‐215. 14655830 10.1007/BF03339978

[148] Veedfald S, Wu T, Bound M, et al Hyperosmolar duodenal saline infusion lowers circulating ghrelin and stimulates intestinal hormone release in young men. Clin Endocrinol Metab. 2018;103(12):4409‐4418. 10.1210/jc.2018-00699 30053031

[149] Kuhre RE, Gribble FM, Hartmann B, et al Fructose stimulates GLP-1 but not GIP secretion in mice, rats, and humans. Am J Physiol Gastrointest Liver Physiol. 2014;306(7):G622‐G630. 24525020 10.1152/ajpgi.00372.2013 PMC3962593

[150] Nauck MA, Quast DR, Wefers J, Meier JJ. GLP-1 receptor agonists in the treatment of type 2 diabetes - state-of-the-art. Mol Metab. 2021;46:101102. 33068776 10.1016/j.molmet.2020.101102 PMC8085572

[151] Nauck MA, D’Alessio DA. Tirzepatide, a dual GIP/GLP-1 receptor co-agonist for the treatment of type 2 diabetes with unmatched effectiveness regrading glycaemic control and body weight reduction. Cardiovasc Diabetol. 2022;21(1):169. 36050763 10.1186/s12933-022-01604-7 PMC9438179

[152] Horowitz M, Aroda VR, Han J, Hardy E, Rayner CK. Upper and/or lower gastrointestinal adverse events with glucagon-like peptide-1 receptor agonists: incidence and consequences. Diabetes Obes Metab. 2017;19(5):672‐681. 28058769 10.1111/dom.12872 PMC5412849

[153] Prasad-Reddy L, Isaacs D. A clinical review of GLP-1 receptor agonists: efficacy and safety in diabetes and beyond. Drugs Context. 2015;4:212283. 26213556 10.7573/dic.212283 PMC4509428

[154] Khan SS, Ndumele CE, Kazi DS. Discontinuation of glucagon-like peptide-1 receptor agonists. JAMA. 2025;333(2):113‐114. 39535741 10.1001/jama.2024.22284

[155] Sisley S, Gutierrez-Aguilar R, Scott M, et al Neuronal GLP1R mediates liraglutide's anorectic but not glucose-lowering effect. J Clin Invest. 2014;124(6):2456‐2463. 24762441 10.1172/JCI72434 PMC4038572

[156] Steinert RE, Beglinger C. Nutrient sensing in the gut: interactions between chemosensory cells, visceral afferents and the secretion of satiation peptides. Physiol Behav. 2011;105(1):62‐70. 21376067 10.1016/j.physbeh.2011.02.039

[157] Krieger JP, Langhans W, Lee SJ. Vagal mediation of GLP-1's effects on food intake and glycemia. Physiol Behav. 2015;152(Pt B):372‐380. 26048300 10.1016/j.physbeh.2015.06.001

[158] Liu F, Wu CG, Tu CL, et al Large library docking identifies positive allosteric modulators of the calcium-sensing receptor. Science. 2024;385(6715):eado1868. 39298584 10.1126/science.ado1868 PMC11629082

